# Tuning the Photoelectrochemical Properties of Ti/W-Modified PCN-222 Using Charge–Selective Interfaces

**DOI:** 10.1021/acsami.5c22732

**Published:** 2026-01-16

**Authors:** Juan Carlos Expósito-Gálvez, Florencia Vattier, José María Pedrosa, Carolina Carrillo-Carrión, Gerko Oskam

**Affiliations:** † Center for Nanoscience and Sustainable Technologies (CNATS). Department of Physical, Chemical and Natural Systems, 16772 Universidad Pablo de Olavide, Seville 41013, Spain; ‡ Department of Inorganic Chemistry, and Center for Innovation in Advanced Chemistry (ORFEO−CINQA). Institute for Chemical Research (IIQ), 16379 CSIC-University of Seville, Seville 41092, Spain; § Institute for Chemical Research (IIQ), CSIC-University of Seville, Seville 41092, Spain

**Keywords:** PCN-222, metal−organic framework (MOF), photoelectrochemical water splitting, hole transport layer (HTL), electron transport layer (ETL)

## Abstract

Metal–organic frameworks (MOFs) have attracted growing interest for photoelectrochemical (PEC) applications, including visible-light photocatalysis, CO_2_ reduction, and hydrogen evolution, owing to their structural tunability and hybrid inorganic–organic nature. The Zr-based porphyrinic framework PCN-222 combines strong visible light absorption from its porphyrin linkers with robust Zr_6_ clusters that act as structural and electronic backbones. Here, we report a modular strategy to tailor and optimize the PEC behavior of PCN-222 through postsynthetic metal-node substitution with Ti, pore encapsulation of phosphotungstic acid (PTA), and integration with charge-selective interfaces. The resulting PCN-222 materials exhibit photoelectrochemical activity across the entire visible range. Whereas pristine PCN-222­(Zr) exhibits photocathodic behavior (photoelectron transfer to the solution and photohole collection at the FTO substrate), partial substitution of Zr with Ti inverts the current to photoanodic. Encapsulation of PTA further enhances the anodic photocurrent due to its electrocatalytic properties. Furthermore, charge-selective TiO_2_ and NiO_*x*_ interlayers deposited between the FTO substrate and the MOF films enable selective extraction of photoelectrons or holes, respectively. This strategy results in a significant photocurrent enhancement, which can be attributed to effective competition of charge extraction and recombination. For PCN-222­(Zr), the cathodic photocurrent increases by a factor of 7 using a NiO_*x*_ interlayer, while the current switches to photoanodic upon TiO_2_ integration, illustrating the importance of efficient charge extraction. Similarly, the current direction is reversed to photocathodic for PCN-222­(Zr/Ti) and PCN-222­(Zr/Ti/W) when using NiO_*x*_. We discuss the interfacial charge extraction, charge transfer and trapping mechanisms in detail, providing strategies for the design of multicomponent MOF-based systems for photoelectrochemical devices.

## Graphical abstract

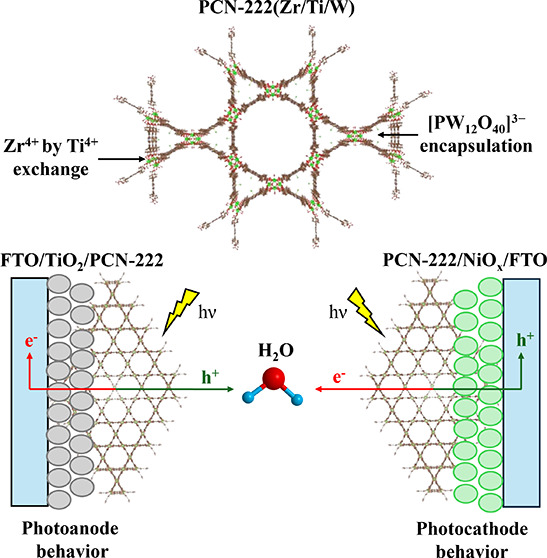

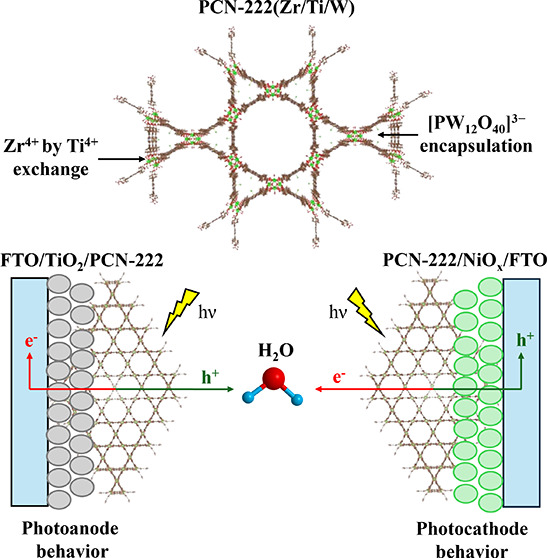

## 1 Introduction

Photoelectrocatalysis (PEC) has been touted as one of the most promising strategies for the synthesis of chemical fuels and value-added products. By coupling light absorption with charge transport and surface redox processes, photoelectrocatalytic systems can drive reactions such as water splitting, CO_2_ reduction, or selective organic oxidation reactions using sunlight as the only energy source. Significant advances have been achieved with inorganic semiconductors, including n-type semiconductors TiO_2_, Fe_2_O_3_, WO_3_ and BiVO_4_, and p-type materials Cu_2_O, CuO and CuBi_2_O_4_; however, these materials often suffer from limited spectral absorption, poor stability (p-type), and limited tunability of their electronic properties.^1,2^ Consequently, a central challenge in PEC research is the rational design of materials that combine broad light absorption with efficient charge separation and transport, while offering structural and chemical flexibility.

Controlling how a photoactive material behaves, whether it acts as an n-type or p-type semiconductor, how its band edges align with the electrolyte redox potentials, and how efficiently charges are transferred through interfacial layers, largely determines the overall PEC performance. The type of semiconductor determines the direction of the photogenerated current: n-type materials typically drive oxidation reactions (photoanodes), while p-type materials favor reduction processes (photocathodes).^3^ Beyond bulk composition, interfacial engineering has proven crucial. Selective charge-transport layers or molecular interlayers can promote directional transport, suppress recombination, and tune band bending at the semiconductor–electrolyte junction.^4,5^ Nevertheless, achieving precise control of such behavior in complex hybrid materials remains a major materials science challenge.

Metal–organic frameworks (MOFs) have emerged over the past decade as a powerful platform to address these issues. MOFs combine crystalline order, chemical modularity, and high surface area, allowing simultaneous control of composition, topology, and functionality.^6^ Their use in photocatalysis and PEC conversion is particularly appealing because both the organic linker and the metal node can contribute to light absorption and charge transport. Pioneering studies have demonstrated that several MOFs, particularly those based on Ti, Fe, or Zr clusters, can display semiconductor-like bandgaps and stable photoactivity in water.^7–10^ The semiconductor behavior of other MOFs, such as MOF-5, was also demonstrated in early studies.^11^ Still, most MOFs are intrinsically insulating, and long-range charge migration is hindered by large intermetallic distances and weak orbital overlap between linkers. Overcoming these limitations through structural design and compositional engineering is therefore an active area of research.

Among all families of photoactive MOFs, porphyrinic frameworks are particularly attractive. The extended π-conjugation of the porphyrin macrocycle affords strong visible-light absorption through Soret and Q bands, while its central metal ion can be exchanged to tailor redox properties.^12^ The archetypal Zr-based PCN-222 exemplifies this concept: it features one-dimensional mesochannels lined with metalloporphyrins and robust Zr_6_O_4_(OH)_4_ nodes that provide exceptional chemical and thermal stability.^13^ PCN-222 and related frameworks have been explored for visible-light photocatalysis, CO_2_ reduction, and hydrogen evolution.^14–17^ Their porphyrinic units act as light harvesters and redox centers, whereas the inorganic nodes serve as electron transport materials and structural supports.

However, despite their favorable optical characteristics, porphyrinic MOFs suffer from rapid charge recombination and poor electronic conductivity across crystals and films. The intrinsic separation between metal clusters and organic linkers hampers delocalization of charge carriers, and the alignment of the frontier orbitals often prevents efficient injection of electrons into external substrates such as FTO.^18^ Considerable effort has therefore been devoted to enhancing the electronic coupling in these systems. Strategies include linker functionalization with donor or acceptor groups^8,14^ ^,^ and incorporation of conductive guests.^19^ However, targeted modification of the inorganic node, either by partial metal substitution or by controlled defect generation, has emerged as the most powerful approach to reshape the electronic landscape and enhance charge transport. Mixed-metal substitution has been successfully employed to tailor band-edge positions and improve electronic coupling in Zr-based MOFs,^20^ while defect engineering strategies have been widely explored to enhance conductivity and catalytic activity in these robust frameworks.^21–23^

Ti-doping or partial substitution of Zr by Ti in PCN-222 is particularly promising because Ti centers possess 3d orbitals of appropriate energy to mediate ligand-to-metal charge transfer (LMCT) processes and enhance electronic communication between porphyrins and the inorganic cluster. In analogy to mixed-metal oxides, introducing Ti into the Zr-oxo cluster can lower the conduction-band edge, improve electron mobility, and enable stronger coupling with conductive supports.^18,20,24^ Moreover, Ti species can act as active sites for interfacial reactions, favoring electron extraction and decreasing the recombination rate at the MOF/FTO interface. Such controlled substitution thus offers a versatile route to modulate the semiconductor character and directionality of the photoresponse.

Complementary to cationic substitution, the incorporation of redox-active molecular guests within MOF pores provides an internal pathway for charge delocalization and temporary electron storage. Polyoxometalates (POMs), and particularly Keggin-type phosphotungstates (PW_12_O_4_^3–^), are well-known electron sponges capable of fast, reversible multielectron reduction while maintaining structural integrity.^25^ When confined inside MOFs, POMs can interact electronically with the framework through hydrogen bonding or coordination to node hydroxyls, forming hybrid materials with synergistic photoactivity.^26^ Encapsulation of an electron-rich POM within MOF matrices and their integration with semiconductor photoanodes has been shown to enhance visible-light-driven charge separation and interfacial electron transfer, thereby improving photocurrent generation and stability in photoelectrochemical water splitting systems.^27^

On the other hand, photoelectrochemical systems exhibit parallel characteristics with third-generation solar cells. For many systems, the solar cell can be considered to consist of an active layer sandwiched between two selective contacts, one for electrons and one for holes. In this case, the charge extraction efficiency at these selective contacts determines the photocurrent rather than the potential distribution.^28–31^ In photoelectrochemical systems, one selective contact is the electrolyte solution, which is generally characterized by slow charge transfer. Hence, efficient extraction of carriers at the FTO contact becomes crucial, which has led to heterojunction systems with the specific goal of improving charge extraction at the FTO contact side. A typical example is the FTO/WO_3_/BiVO_4_ system, where WO_3_ functions as a selective contact with a high electron extraction rate, thus significantly diminishing recombination in the BiVO_4_ absorber layer.^32,33^

These observations highlight how subtle modifications, either intrinsic (via Ti substitution or POM incorporation) or extrinsic (through interfacial contacts), may provide opportunities to tailor the operational mode of a porphyrinic MOF electrode. Within this broad context, the present work investigates the interplay between composition, electronic structure, and photoelectrochemical characteristics in thin films of the porphyrinic MOF PCN-222­(Zr), with a special emphasis on the influence of charge-selective interlayers on their performance. Three representative systems were fabricated on FTO (fluorine-doped tin oxide) substrates (or FTO substrates incorporating interfacial transport layers): pristine PCN-222­(Zr), a mixed-metal analogue with partial substitution of Zr by Ti PCN-222­(Zr/Ti), and a subsequent composite containing encapsulated phosphotungstic acid, H_3_PW_12_O_40_ (PTA), PCN-222­(Zr/Ti/W). By combining UV–vis spectroscopy, transient photocurrent, and linear-sweep voltammetry under chopped illumination, we analyze how each structural modification alters the optical response, charge-separation efficiency, and directionality of the photocurrent. The results reveal that Ti incorporation and subsequent H_3_PW_12_O_40_ encapsulation collectively shift the photoelectrode behavior from photocathodic to photoanodic under visible-light illumination, with a strong dependence on the nature of the interfacial layer. Together, these findings provide fundamental insight into how rational chemical modification of MOF nodes, pores, and interfaces can tailor semiconductor behavior in hybrid photoelectrodes, bridging the gap between molecular light-harvesting units and extended solid-state architectures.

## 2 Experimental Section

### 2.1 Synthesis of PCN-222­(Zr) Nanoparticles

Nanoparticles of PCN-222 were synthesized in an Initiator Classic Microwave reactor (Biotage) by using a microwave-assisted (MW) method previously optimized in our group.^34^ First, the Zr_6_ clusters, [Zr_6_(μ_3_-O)_4_(μ_3_–OH)_4_], were prepared according to a previously reported method.^35^ For the nanoparticle synthesis, 76 mg (28.4 μmol) of Zr_6_ clusters and 22.5 mg (28.5 μmol) of tetrakis­(4-carboxyphenyl)­porphyrin (BD168635, BLD Pharmatech) were dissolved in 8 mL of anhydrous *N*, *N*-dimethylformamide (DMF) (481785, Panreac) and placed into a 10 mL MW vial. Next, 100 μL of trifluoroacetic acid (302031, Sigma-Aldrich) was added to the vial. The mixture was subjected to an initial MW power of 250 W, reaching 100 °C within minutes, and further maintained for 10 min. After this time, the reaction was rapidly quenched by forced air cooling to suppress further crystal growth. The obtained nanoparticles were collected by centrifugation (10,000 RCF, 15 min), washed twice with fresh DMF and twice with methanol (131091, Panreac). The purified product was redispersed in methanol to obtain a 5 mg mL^–1^ suspension, which was stored at 4 °C until use.

### 2.2 Preparation of PCN-222­(Zr/Ti) Nanoparticles: Metal-Exchange at the Nodes

The incorporation of Ti into PCN-222 nanoparticles was performed using a newly optimized microwave-assisted method aimed at maximizing the amount of Ti incorporated. The previously prepared PCN-222 was dried and thermally activated in an oven at 120 °C for 6 h before performing the postsynthetic modification (PSM) to introduce Ti into the structure. For this purpose, 20 mg (8.5 μmol) of activated PCN-222 and 10 mg (40 μmol) of bis­(cyclopentadienyl)­titanium­(IV) dichloride (TiCp_2_Cl_2_) (234826, Sigma-Aldrich) were dissolved in 6 mL of anhydrous DMF and transferred into a 10 mL MW vial. The mixture was heated to 150 °C by MW irradiation and maintained at this temperature for 30 min. After this period, the modified PCN-222­(Zr/Ti) nanoparticles were collected by centrifugation (10,000 RCF, 15 min), washed twice with fresh DMF and twice with methanol. The purified product was redispersed in methanol to obtain a 5 mg mL^–1^ suspension, which was stored at 4 °C until use.

### 2.3 Preparation of PCN-222­(Zr/Ti/W) Nanoparticles: Encapsulation of H_3_PW_12_O_40_ into the Pores

PCN-222 was dried and thermally activated in an oven at 120 °C for 6 h before performing the PSM to load H_3_PW_12_O_40_ molecules into the pores of the framework via an impregnation method under mild temperature conditions. Specifically, 20 mg (8.5 μmol) of activated PCN-222 and 80 mg (27.8 μmol) of phosphotungstic acid hydrate (79690, Sigma-Aldrich) were dissolved in 6 mL of anhydrous DMF and placed into a 10 mL microwave vial. The mixture was allowed to soak for 30 min and then incubated at 60 °C under continuous stirring for 48 h. Afterward, the modified PCN-222­(Zr/Ti/W) nanoparticles were collected by centrifugation (10,000 RCF, 15 min), washed three times with methanol, and finally redispersed in methanol to obtain a 5 mg mL^–1^ suspension. The sample was stored at 4 °C until use.

### 2.4 Photoelectrode Fabrication

The FTO-coated conductive glass substrates (SnO_2_:F, 12–15 Ω sq^–1^, TEC 15 Xop Glass) were sequentially cleaned using an ultrasonic bath for 20 min in each of the following solvents: Milli-Q water containing Hellmanex III (Z805939, Sigma-Aldrich), Milli-Q water, ethanol (20821, VWR), isopropanol (278475, Sigma-Aldrich), and acetone (20067, VWR). After solvent cleaning, the substrates were dried using a nitrogen stream and subsequently treated in a UV ozone cleaner (Ossila, L2002A3-EU) for 30 min at room temperature.

TiO_2_ and NiO_*x*_ layers were deposited on FTO substrates via spray pyrolysis with the substrate at 300 °C under N_2_ flow. The precursors were 0.14 M titanium diisopropoxide bis­(acetylacetonate) (325252, Sigma-Aldrich) in isopropanol and 0.05 M nickel­(II) nitrate hexahydrate (203874, Sigma-Aldrich) in water, respectively. A total of 20 and 15 layers were deposited, respectively, with a 30 s interval between each deposition step. After reaching the desired number of layers, the films were kept at 300 °C for 30 min and allowed to cool to room temperature.

The photoelectrodes were prepared by depositing 20 μL of a 5 mg mL^–1^ PCN-222­(Zr, Zr/Ti, and Zr/Ti/W) previously sonicated dispersion in methanol onto FTO, FTO/TiO_2_, and FTO/NiO_*x*_ substrates via a two-step spin-coating process: first at 500 rpm for 10 s to ensure covering the FTO substrate, followed by 3000 rpm for 30 s. This procedure was repeated for a total of 3 sequential deposition steps, with each layer left to dry at room temperature for 5 min to ensure solvent evaporation before the next coating step (see Figure S1).

### 2.5 Structural, Morphological, and Optical Characterization

Scanning electron microscopy (SEM) to evaluate the morphology and homogeneity of the nanoparticles was performed on a HITACHI S4800 field emission microscope operating at 2 kV. Samples were prepared by drying a diluted suspension of the particles in methanol on a silicon wafer substrate. Elemental composition of the nanoparticles (e.g., relative metal ratios Ti/Zr or W content) was estimated by semiquantitative energy dispersive X-ray analysis (EDX) using the same SEM instrument coupled to a Bruker-X Flash-4010 EDX detector and operating at 10 kV.

Powder X-ray diffraction (PXRD) of the crystalline powder of the samples was performed using a Bruker D8-Advance Diffractometer. Cu Kα X-ray radiation was used and the measurement range was 1.5°–30° (2θ) with a step of 0.02°. Grazing-angle X-ray diffraction (GAXRD) was performed using an incidence angle of 2°, in the 2θ range 2°–60° with a step size of 0.01° per 0.2 s was used to determine the crystalline structure of the PCN-222 films. All measurements were collected in 2D mode using a Bruker EIGER2 R 250 K Detector.

Dynamic light scattering (DLS) measurements on the nanoparticles in suspension (in methanol) were carried out using a Malvern Zetasizer Nano ZSP equipped with a 10 mW He–Ne laser operating at a wavelength of 633 nm and fixed scattering angle of 173̊. The ζ-potential of the nanoparticles dispersed in water was measured with laser Doppler anemometry (LDA) using the same Malvern Zetasizer Nano ZSP instrument.

X-ray photoelectron spectroscopy (XPS) spectra were recorded in a Phoibos-100 spectrometer with a nonmonochromatized X-ray source (Al Kα; 1486.6 eV) and the power source was 230 W. The electron energy hemispherical analyzer was operated in the constant pass energy mode. Low-resolution survey spectra were obtained with a pass energy of 50 eV, while high-energy resolution spectra of detected elements were obtained with a pass energy of 20 eV. The spectra were analyzed with the “CASA XPS” software (version 2.3.16.Dev52). Binding energies were calibrated using C 1s signal as an internal standard at 284.8 eV. Shirley-type backgrounds were used to determine the areas under the peaks.

The homogeneity, film thickness, and surface morphology were examined in both top-view and cross-section modes using a Zeiss Gemini 300 field emission scanning electron microscope (FE-SEM), with an accelerating voltage of 15 kV. Energy-dispersive X-ray spectroscopy (EDX) was used to estimate the elemental distribution of photoelectrode materials using the same FE-SEM system. The films were previously coated with a 10 nm gold layer using a Vac Coat sputtering system (model DSCT) to improve surface conductivity and minimize charging effects during SEM measurements.

The absorbance spectrum of a diluted suspension (0.03 mg mL^–1^) of PCN-222 in methanol was measured using an Agilent UV–vis Cary 100 spectrophotometer (model G9821A), in the range of 350–800 nm. To determine the optical properties of the films, diffuse reflectance spectroscopy (DRS) was performed using a UV/vis/NIR FSL1000-DD-STM Edinburgh Instruments spectrophotometer equipped with an integrating sphere and an Xe lamp as the light source in the 350–800 nm range, with a dwell time of 1 s and step size of 1 nm.

The total reflectance *R*(λ) was calculated as *R*(λ) = *R*_sample_(λ)/*R*_reference_(λ), where *R*_reference_(λ) corresponds to the bare FTO reflectance. Since the integrating sphere captures both the specular and diffuse components, this measurement accounts for the overall reflected light. In this configuration, the sample was placed over a diffusely reflective white holder (same material as the sphere interior), which redirects the transmitted light back toward the sample. As a result, any light that is not ultimately reflected into the sphere is considered absorbed. Under these conditions, the absorptance can be estimated as *A*(λ) = 1 – *R*(λ), which corresponds to the light-harvesting efficiency (*LHE*) of the films (i.e., *LHE*(λ) ≈ *A*(λ)), representing the fraction of absorbed incident photons at each wavelength.

### 2.6 Photoelectrochemical Characterization

Photoelectrochemical (PEC) measurements were carried out using a three-electrode setup with front-side illumination (i.e., from the electrolyte side) in a single-compartment electrochemical cell equipped with a quartz window. A Pt wire served as the counter electrode, while an Ag/AgCl electrode (3 M KCl) (P3911, Sigma-Aldrich) was used as the reference. The electrolyte was a 0.1 M acetate buffer (1.06267, Supelco and 211008, Panreac) solution at pH 4.5, which was purged with N_2_ for 20 min before the measurement to remove dissolved gases, such as O_2_. Measurements were performed with an Autolab PGSTAT302N system and a solar simulator (ABET 1100) equipped with an AM 1.5G filter, calibrated to 1 sun (100 mW cm^–2^) using a silicon photodiode (Newport model 91150), and under high-power monochromatic LED illumination of different wavelengths: UV (λ = 400 nm), blue (λ = 455 nm), green (λ = 535 nm) and red (λ = 670 nm) calibrated with a Si-photodiode (Hamamatsu S12698–2) at the same photon flux of 1.6 × 10^16^ cm^–2^ s^–1^. Both for the solar simulator and LED illumination, a chopper was used to modulate the light intensity at a frequency of 0.18 Hz. The emission spectra of the light-emitting diodes (LEDs) were obtained using an Ocean Optics (USB 4000) spectrometer equipped with an optical fiber. The applied potential was converted to the reversible hydrogen electrode (RHE) scale using the equation: *E*(vs RHE) = *E*(vs Ag/AgCl) + 0.21V + 0.0591 × pH, where 0.21 V corresponds to the potential of Ag/AgCl (3 M KCl) versus the normal hydrogen electrode (NHE). The illuminated area in contact with the electrolyte was 1 cm^2^ and the scan rate was 20 mV s^–1^. The photoelectrochemical characteristics remained unchanged over multiple days and numerous measurements.

A solution of 0.1 M acetic acid/sodium acetate buffer with 0.01 M [Fe­(CN)_6_]^4–^ (P3289, Sigma-Aldrich) and other with 0.01 M [Fe­(CN)_6_]^3–^ (26810, VWR) both at pH = 4.5, and a nonaqueous solution 0.1 M TBAPF_6_ (86874, Sigma-Aldrich) and 0.01 M *p*-benzoquinone (A13162, Thermo Scientific) in propylene carbonate (310328, Sigma-Aldrich) under dark conditions were used to verify the compactness of the TiO_2_ and NiO_*x*_ charge-selective layers, respectively.

## 3 Result and Discussion

### 3.1 Synthesis and Structural Characterization of Ti/W-Modified PCN-222

In a first step, PCN-222­(Zr/Ti) was obtained by a postsynthetic metal-node exchange using a newly developed method, not previously reported for the incorporation of Ti into MOFs. It consists of employing TiCp_2_Cl_2_ in DMF under controlled microwave heating (see Experimental Section for details) to take advantage of microwave irradiation, achieving high incorporation efficiency while drastically reducing reaction times from several days, as typically reported in the literature, to only 30 min^36^ The method relies on the high hydrolytic and thermal stability of PCN-222­(Zr) and on the similar coordination chemistry of Zr­(IV) and Ti­(IV), enabling an efficient exchange of Zr by Ti without compromising the structural integrity of the material. Thus, this postsynthetic modification strategy also overcomes one of the main challenges associated with the direct synthesis of Ti-based MOFs, namely the severe hydrolysis of most Ti precursors and the poor reversibility of Ti–ligand coordination, which often prevents the formation of well-defined crystalline frameworks. Following Ti incorporation, the color of the material changes from purple to brown (Figure 1a), a transformation attributed to perturbations in the electronic structure induced by metal substitution within the metal nodes, a phenomenon previously rationalized and proposed as a strategy for tuning the electronic structure in MOFs.^20^ SEM reveals that the characteristic nanorod morphology of PCN-222 is largely preserved (Figure 1b), with an average lateral dimension of approximately 180 nm. Some changes in aspect ratio and surface texture are observed, likely as a consequence of the intense MW heating applied. In particular, the slightly roughened surface may indicate the formation of structural defects or the presence of localized Ti-rich domains at the particle surface. Reducing the MW heating temperature to 100 °C prevented such morphological alterations but also markedly decreased the Ti incorporation efficiency. Therefore, MW heating at 150 °C for 30 min was selected as the optimal condition, maximizing Ti incorporation while preserving acceptable morphological characteristics.

**Figure 1:**
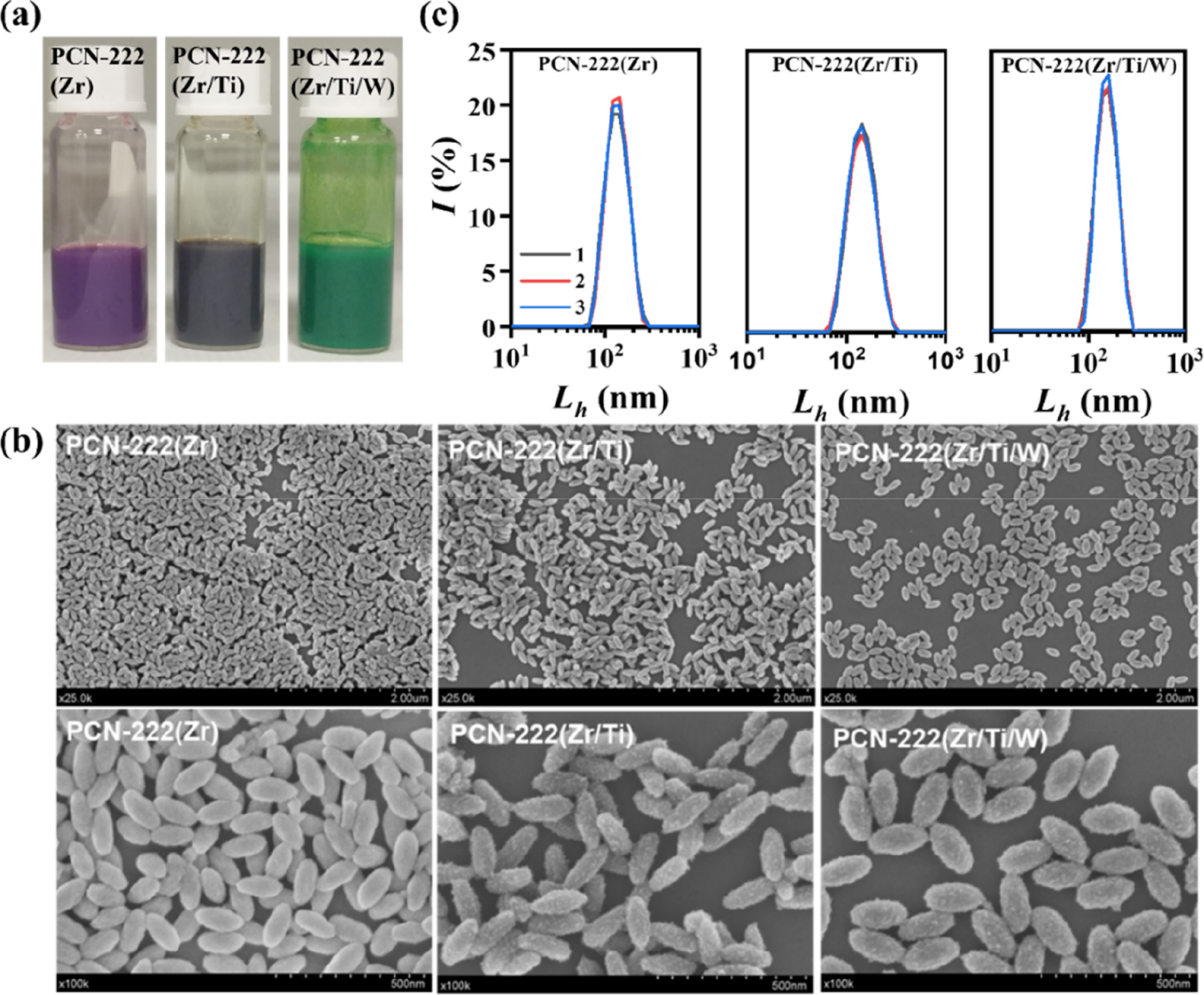
(a) Photographs of the as-prepared samples: PCN-222­(Zr), PCN-222­(Zr/Ti), and PCN-222­(Zr/Ti/W) dispersed in methanol. (b) SEM images at different magnifications of pristine PCN-222­(Zr) nanoparticles and after Ti/W modifications. (c) DLS size distribution by intensity of the as-prepared PCN-222 nanoparticles dispersed in methanol (*n* = 3 measurements).

In a second step, the well-defined mesoporosity of PCN-222 was exploited to encapsulate a Keggin-type polyoxometalate species (POM), specifically H_3_PW_12_O_40_, into the framework cavities via a straightforward impregnation method using methanol (see Experimental Section), yielding the composite material denoted as PCN-222­(Zr/Ti/W). Given the size mismatch between the ≈3.6 nm mesochannels of PCN-222 and the ≈1 nm Keggin anion, PTA incorporation is expected to only partially occupy the pore volume. The [PW_12_O_40_]^3–^ clusters are strongly retained within the mesopores due to favorable electrostatic interactions with the positively charged Zr/Ti–oxo nodes and coordinated aquo/hydroxo ligands at the metal clusters, as previously reported for other MOF systems, specifically NU-1000.^37^ SEM images of the resulting PCN-222­(Zr/Ti/W) nanoparticles reveal minimal morphological changes, with a slight variation in the aspect ratio of the nanorods, which appear more rounded. The color change to green observed after encapsulation of H_3_PW_12_O_40_ (Figure 1a) arises from the characteristic optical properties of the Keggin-type polyoxometalate. When confined within the MOF structure, additional ligand-to-metal charge-transfer (LMCT) transitions, particularly from oxygen to tungsten (O → W) are induced, leading to enhanced absorption in the visible region and giving the material its distinctive green color. This behavior aligns with prior reports, where photochromic effects have been observed after incorporating a Keggin anion into UiO-66 and UiO-67 frameworks, attributed to charge-transfer effects and guest-induced band modulation.^38,39^

The atomic composition of the modified PCN-222 materials was estimated by EDX (semiquantitative) (Table S1), supporting efficient incorporation of Ti and W into the structure. For PCN-222­(Zr/Ti), the Ti content was 48% relative to the total (Zr + Ti) atomic fraction, indicating substantial Zr-to-Ti exchange within the clusters. Notably, the achieved Ti-incorporation value is considerably higher than that reported for Ti-doped PCN-224 after 48 h of reaction, which reached only ∼36%.^36^ In the fully modified PCN-222­(Zr/Ti/W) sample, the Ti content was 46% of the total metal content (Ti/Zr = 0.88), showing that the subsequent Keggin-type polyoxometalate [PW_12_O_40_]^3–^ encapsulation did not displace the incorporated Ti. The W content corresponds to 0.6 PW_12_ units per Zr_6_ node, which is close to the theoretical maximum expected for full occupation of the mesopores (based on geometric constraints and pore volume analysis of PCN-222). The high encapsulation level achieved here reflects both the strong electrostatic affinity between the anionic POM clusters and the cationic metal centers in the PCN framework, as well as the accessibility of the large mesoporous channels. Comparable high loadings have been reported for NU-1000-based systems, typically in the range of 0.6–0.8 PW_12_/Zr_6_ node.^37,40^ These compositional estimates are consistent with the structural (PXRD) and chemical-state (XPS) signatures discussed below, which support mixed Zr/Ti-oxo node formation and PTA incorporation.

DLS analysis of the as-prepared nanoparticles dispersed in methanol reveals only slight changes in the hydrodynamic size after Ti- and W- modifications (Figure 1c and Table S2), consistent with the SEM observations. The moderate increase observed for PCN-222­(Zr/Ti/W) may be attributed to slight particle aggregation induced by POM encapsulation. Importantly, the polydispersity index (PDI) values remain low after both PSM steps (PDI ≤ 0.1), indicating good particle uniformity and the absence of aggregation. Zeta-potential measurements show a slight increase in surface positive charge following Ti-incorporation (from +27.2 mV for PCN-222­(Zr) to +34.6 mV for PCN-222­(Zr/Ti), likely due to the formation of Ti-rich domains at the particle surface. The subsequent encapsulation of [PW_12_O_40_]^3–^ species produces only a very minimal change in surface charge (Table S2), supporting the conclusion that these species are mainly confined within the internal mesopores rather than adsorbed onto the particle surface. Overall, PTA incorporation reduces the free pore volume, but the mesoporous channels remain sufficiently open to allow electrolyte access and ionic transport under the photoelectrochemical conditions used in this work.

The PXRD pattern of pristine PCN-222 matches well with the simulated pattern (Figure 2a). After Ti-incorporation via metal-node exchange, the diffraction peak at 2θ ≈ 2.4°, assigned to the (100) crystallographic plane, exhibits a clear splitting. This effect suggests lattice distortion induced by the substitution of Zr­(IV) by the smaller Ti­(IV) ion, potentially lowering the framework symmetry or giving rise to coexisting structural domains. Similar PXRD peak splitting in MOFs has previously been attributed to symmetry reduction and framework distortion, as reported for the NTU-9 MOF.^41,42^ The (200) reflection at 2θ–4.8° also shows splitting along with a marked decrease in intensity, which can be ascribed to increased microstrain, reduced crystallographic coherence, and/or compositional heterogeneity introduced in the PCN-222­(Zr/Ti) sample. In contrast, the PXRD pattern of the PCN-222­(Zr/Ti/W) reveals a notable increase in the intensity of the (211) and (201) reflections (at 2θ–6.65° and 7.06°, respectively). This enhancement arises from the extra electron density of [PW_12_O_40_]^3–^ species confined within the mesopores, which increases the X-ray scattering contrast along specific crystallographic directions.^40^ In this case, the selective intensification of these reflections supports the successful and uniform encapsulation of the [PW_12_O_40_]^3–^ guests while preserving the structural integrity of the host framework.

**Figure 2:**
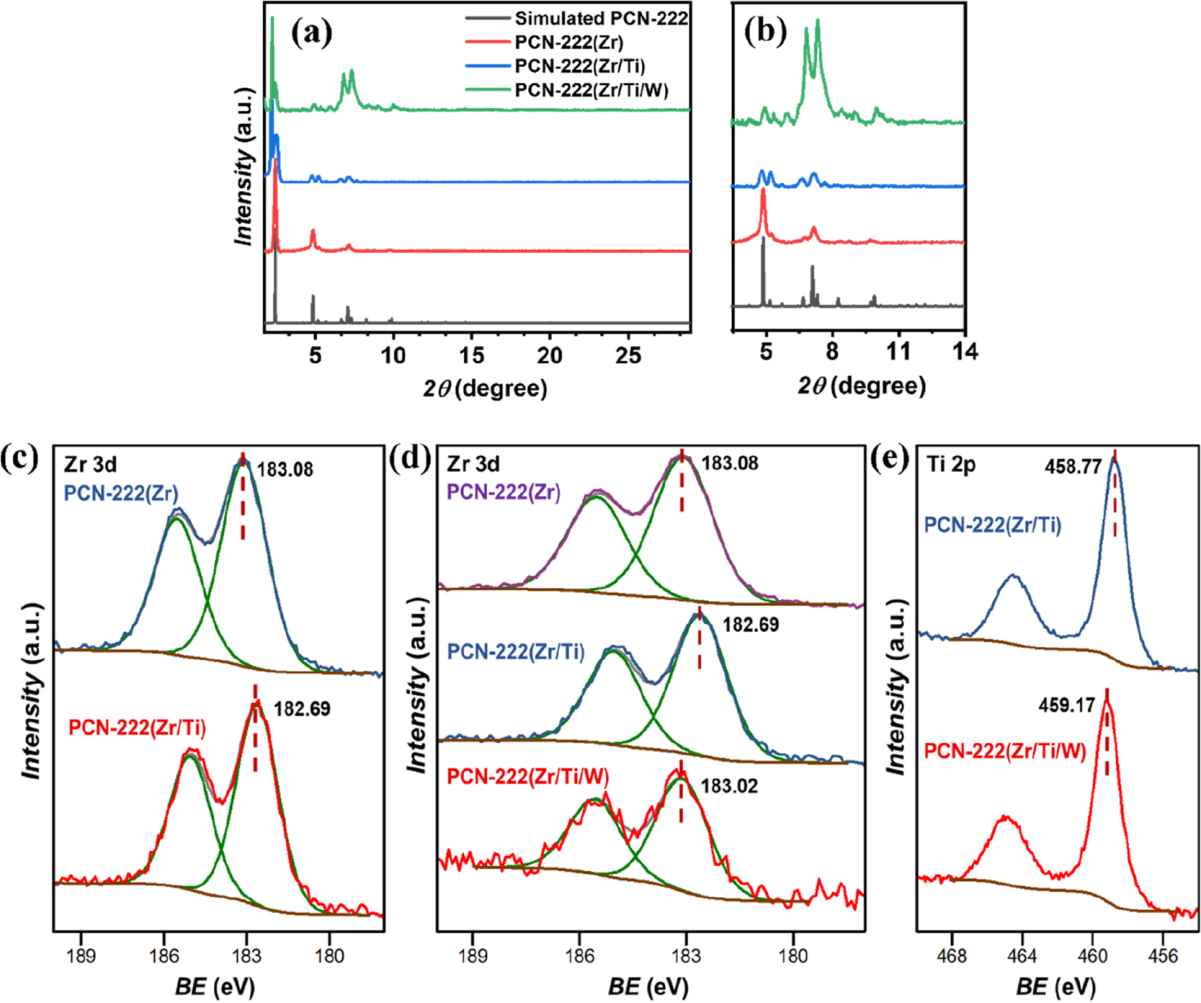
(a) PXRD patterns of the as-prepared nanoparticles, including the simulated pattern for PCN-222 (CCDC 893545), within the range of (a) 2θ = 1.7–29°, and (b) magnification of the 2θ = 3.5–14° region to more clearly visualize the changes. (c) Zr 3d XPS spectra of PCN-222­(Zr) (top, blue) and PCN-222­(Zr/Ti) (bottom, red). (d) Comparison of Zr 3d XPS spectra of PCN-222­(Zr) (top, purple), PCN-222­(Zr/Ti) (middle, blue) and PCN-222­(Zr/Ti/W) (bottom, red). (e) Comparison of Ti 2p XPS spectra of PCN-222­(Zr/Ti) (top, blue) and PCN-222­(Zr/Ti/W) (bottom, red).

XPS analyses were carried out to investigate the surface composition and chemical states of the as-prepared nanoparticles. Figure S2a displays the high-resolution spectra of the Zr 3d, O 1s, and C 1s regions for PCN-222­(Zr) nanoparticles. The Zr 3d spectrum exhibits two peaks at binding energies (BE) of 183.08 and 185.51 eV, with a spin–orbit splitting (SOS) of 2.43 eV, corresponding to Zr 3d_5/2_ and Zr 3d_3/2_, respectively. These values are characteristic of Zr­(IV) cations within Zr_6_ clusters commonly observed in this class of MOFs.^43,44^ The O 1s spectrum was deconvoluted into three components located at 531.15, 532.10, and 533.61 eV, assigned to Zr–O–Zr, Zr–O–C, and Zr–OH environments, respectively, with the central peak being the dominant contribution.^45^ The C 1s region was well fitted with four components at 284.77, 285.49, 288.82, and 292.67 eV, attributed to C=C (aromatic), C–O, O–C=O, and a shakeup satellite feature, respectively.^45^ The N 1s signal appears as a broad feature centered at 399.95 eV, consistent with the porphyrinic nitrogen environment (Figure S2c).

The successful incorporation of Ti into PCN-222­(Zr/Ti) is confirmed by the Ti 2p doublet (Figures 2e and S2b) at 458.77 eV (Ti 2p_3/2_) and 464.31 eV (Ti 2p_1/2_). The observed spin–orbit splitting (SOS) of 5.54 eV, along with the BE positions and full width at half-maximum (fwhm) values, is consistent with the Ti­(IV) oxidation state, as expected for materials prepared under ambient conditions. The Zr 3d signal shows peaks at 182.69 eV (Zr 3d_5/2_) and 185.12 eV (Zr 3d_3/2_) BE. Notably, a negative shift in binding energy (ΔBE = −0.39 eV) was observed, compared to PCN-222­(Zr), Figure 2c. This shift reflects a modified local electronic environment at the Zr centers upon Ti incorporation, consistent with a reduced electron-withdrawing character within the Zr_6_ clusters when Ti­(IV) is introduced, leading to the formation of mixed Zr/Ti-oxo nodes.^43,45,46^ In the O 1s region, two main components at 532.31 and 530.38 eV are resolved and assigned to Zr–O and Ti–O bonds, respectively, further supporting the formation of mixed-metal oxo nodes. The N 1s signal remains essentially unchanged (400.05 eV), indicating that the chemical environment of the porphyrinic nitrogen atoms is unaffected by Ti incorporation (Figure S2c). These findings indicate that Ti atoms are not coordinated to the porphyrinic macrocycle linkers but are instead incorporated into the inorganic nodes, resulting in mixed Ti–Zr clusters.^47^ Notably, these XPS signatures correlate with the structural distortions inferred from PXRD (shown in Figure 2a), where the splitting of the (100) reflection at 2θ–2.4° and the concomitant changes at the (200) reflection (2θ–4.8°) point to symmetry lowering and microstrain in the framework. The chemical shift of Zr 3d toward lower BE and the emergence of a distinct Ti–O contribution in O 1s are fully consistent with such lattice perturbations and with the coexistence of Zr-rich and Ti-perturbed local environments at the nodes, an electronic/structural heterogeneity that rationalizes the PXRD peak splitting previously discussed for PCN-222­(Zr/Ti).

For the PCN-222­(Zr/Ti/W) sample, the survey spectrum confirmed the presence of P and W elements along with Zr, Ti, O, N, and C. The high-resolution W 4f spectrum (Figure S2d) exhibits two peaks at 35.90 and 38.08 eV BE, assigned to W 4f_7/2_ and W 4f_5/2_, with a SOS of 2.18 eV, consistent with the W­(VI) oxidation state of encapsulated H_3_PW_12_O_40_ species.^48^ The Zr 3d region spectrum (Figure 2d) shows two peaks at 183.02 and 185.45 eV BE, corresponding to the Zr 3d_5/2_ and Zr 3d_3/2_ photoemission. These values are slightly higher than those recorded for PCN-222­(Zr/Ti), likely due to the strong electron-withdrawing effect of the PTA moieties, which reduce the electron density around the Zr centers. Similarly, the Ti 2p peaks shifted to higher binding energies (459.17 and 464.71 eV), reflecting the same effect (Figure 2e). Finally, the O 1s spectrum displays two components at 532.55 eV (Zr–O) and 530.93 eV (Ti–O), while the N 1s peak remained almost unchanged at 400.60 eV (400.42 eV in PCN-222­(Zr/Ti/W)), indicating preservation of the porphyrinic fragment. These electronic shifts observed by XPS correlate with PXRD observations. The increased intensity of the (111) and (201) reflections in PCN-222­(Zr/Ti/W) (Figure 2a) is consistent with the higher electron density introduced by [PW_12_O_40_]^3–^ encapsulation. Together, the data confirm strong interaction of the POM with the metal–oxo nodes, which perturbs the electronic structure while preserving the crystallinity of the framework.

Based on these characterization results, we can conclude that the proposed stepwise PSM approach, taking full advantage of the structural robustness, chemical tunability, and hierarchical porosity of PCN-222, enables dual-site functionalization at both the metal nodes and the mesopores, thereby creating multicomponent hybrid MOF materials.

### 3.2 Characterization of the Photoelectrodes Containing the Active Ti/W-Modified PCN-222

The deposition process of the Ti/W-modified PCN-222 materials onto FTO substrates as well as FTO covered with the charge-selective interfacial layers, FTO/TiO_2_ and FTO/NiO_*x*_, was optimized for the fabrication of reproducible photoelectrodes. SEM images of PCN-222­(Zr) spin-coated film on FTO are shown in Figure 3a (top-view) and Figure 3b (cross-section), revealing a homogeneous layer with complete coverage of the FTO substrate, and an average thickness of 1.1 μm. Similar results were obtained for PCN-222­(Zr/Ti) and PCN-222­(Zr/Ti/W). EDX analysis confirms a uniform distribution of the elements comprising PCN-222 (i.e., O, C, and Zr), as well as the presence of Ti and W in the samples where these metals were incorporated into the PCN-222 particles (see Figures S3–S5). Figures S6 and S7 reveal that Ti and Ni are the only detected elements, apart from O, in the FTO/TiO_2_ and FTO/NiO_*x*_ thin layers, respectively, along with Sn from the FTO substrate. Figure 3c displays the GAXRD patterns of the PCN-222 electrode, showing the characteristic peak at 2θ–2.4° corresponding to the (100) plane of PCN-222 (CCDC 893545).

**Figure 3:**
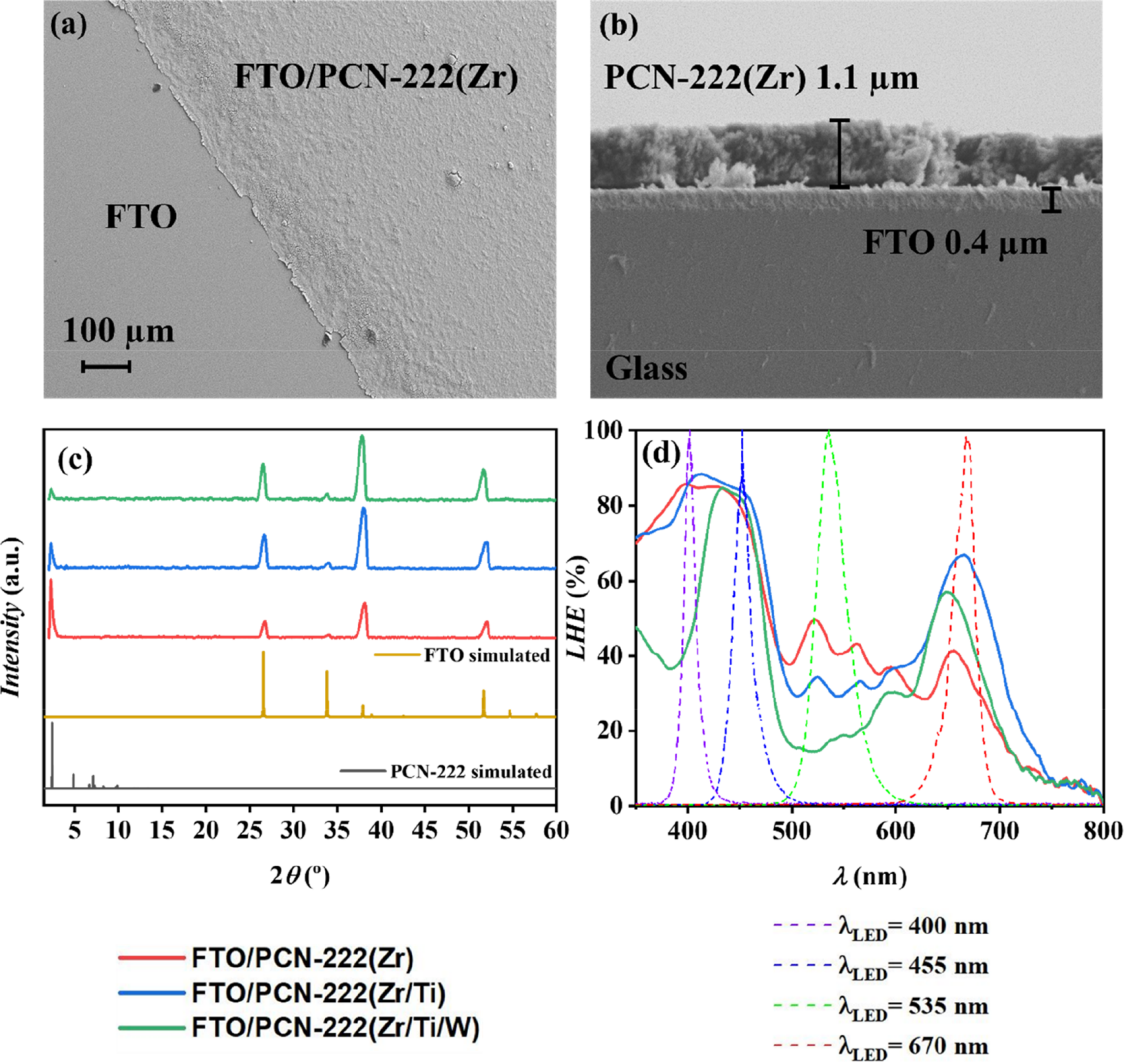
(a) SEM top-view image, and (b) cross-section image of FTO/PCN-222­(Zr) photoelectrode. (c) GAXRD diffractograms of spin-coated PCN-222 on FTO and simulated patterns for comparison (FTO: COD 1534785 and PCN-222: CCDC 893545). (d) LHE spectra (lines) of FTO/PCN-222 and illumination spectra (dashed lines) of each LED used in photoelectrochemical measurements.

The intensity of this peak decreases upon incorporation of Ti, and further with Ti/W. It should be noted that the absolute peak intensities in these GAXRD patterns (thin films on FTO, at an incidence angle of 2°) are not directly comparable to the powder PXRD patterns in Figure 2a, because they are strongly influenced by the effective diffracting volume (film thickness/areal mass), preferred orientation, surface roughness, and the background/scattering contribution from the FTO substrate, particularly at low angles. Therefore, the lower (100) peak height observed for FTO/PCN-222­(Zr/Ti/W) reflects the combined effects of thin-film measurement conditions and peak broadening/microstrain upon Ti/W modification, rather than a loss of the PCN-222 structure. The remaining peaks at 26.5°, 33.8°, 37.9°, and 51.7° are attributed to the FTO substrate (COD 1534785). In the case of FTO/TiO_2_ and FTO/NiO_*x*_, the layers were too thin to yield reliable XRD data or accurate thickness measurements; however, this did not affect the optical properties of the film.

Figure S8 shows the absorbance spectra of both dispersed and FTO-deposited PCN-222 samples. In dispersion (Figure S8a), the characteristic Soret band appears at 412 nm, along with the four Q-bands at 510, 545, 585, and 640 nm.^49,50^ Upon deposition on FTO (Figure S8b–e), a red-shift is observed, 7 nm for PCN-222­(Zr), 21 nm for PCN-222­(Zr/Ti), and 34 nm for PCN-222­(Zr/Ti/W), accompanied by a peak broadening, as evidenced by a 20 nm increase in the full-width-at-half-maximum (fwhm). These spectral changes can be attributed to several factors: first, the red-shift may arise from π–π interactions between neighboring PCN-222 self-assembled during film formation, which leads to excitonic coupling and lowers the energy of the electronic transitions. Second, changes in the coordination environment of the PCN-222 once deposited or changes in the local dielectric environment at the PCN–FTO interface can influence the energy and width of the Soret band. Notably, this red-shift is significantly more pronounced for the Ti- and Ti/W-modified samples; however, the corresponding fwhm values remain comparable across all samples. This observation suggests that dielectric effects at the PCN–FTO interface, rather than particle assemblies, are the dominant factor governing the magnitude of the observed spectral shifts. In the case of the dispersed PCN-222­(Zr/Ti/W), two peaks are observed in the Soret band region; the first corresponds to the Soret absorption of the porphyrin units from well-dispersed MOF particles, while the second arises from the characteristic absorption of the encapsulated H_3_PW_12_O_40_ species within the mesopores. Note that H_3_PW_12_O_40_ in aqueous solution displays two absorption bands centered at 355 and 480 nm^51^

As a result of the red-shift of the optical transitions observed for the Ti- and Ti/W-modified samples relative to pristine PCN-222­(Zr), a slight reduction in the bandgap energy occurs. For PCN-222­(Zr), (Zr/Ti), and (Zr/Ti/W), the estimated optical bandgaps in dispersion were 1.89, 1.89, and 1.82 eV, respectively. After film formation, independent of whether the analysis is for indirect (Figure S9) or direct (Figure S10) transitions, decreased to ≈1.75 eV for all photoelectrodes due to interparticle interactions, light scattering, and morphological effects in films. These values were estimated using both the Tauc plot analysis (from the absorbance spectra in Figure S8) and Kubelka–Munk analysis (from the diffuse reflectance spectra in Figure S11).^52,53^ Figure 3d shows the LHE values (obtained from Figure S11) of the FTO/PCN-222 photoelectrodes and the illumination spectra of the monochromatic LEDs used for photoelectrochemical measurements.

### 3.3 Tailoring the Photoelectrochemical Activity in PCN-222-Based Electrodes

Figure 4 illustrates linear sweep voltammetry (LSV) curves for FTO/PCN-222­(Zr), FTO/PCN-222­(Zr/Ti), and FTO/PCN-222­(Zr/Ti/W) photoelectrodes compared with bare-FTO in 0.1 M acetate buffer as electrolyte at a scan rate of 20 mV s^–1^ with chopped front side illumination using different monochromatic LED wavelengths: UV (λ = 400 nm), blue (λ = 455 nm), green (λ = 535 nm) and red (λ = 670 nm) at the same photon flux of 1.6 × 10^16^ cm^–2^ s^–1^.

**Figure 4:**
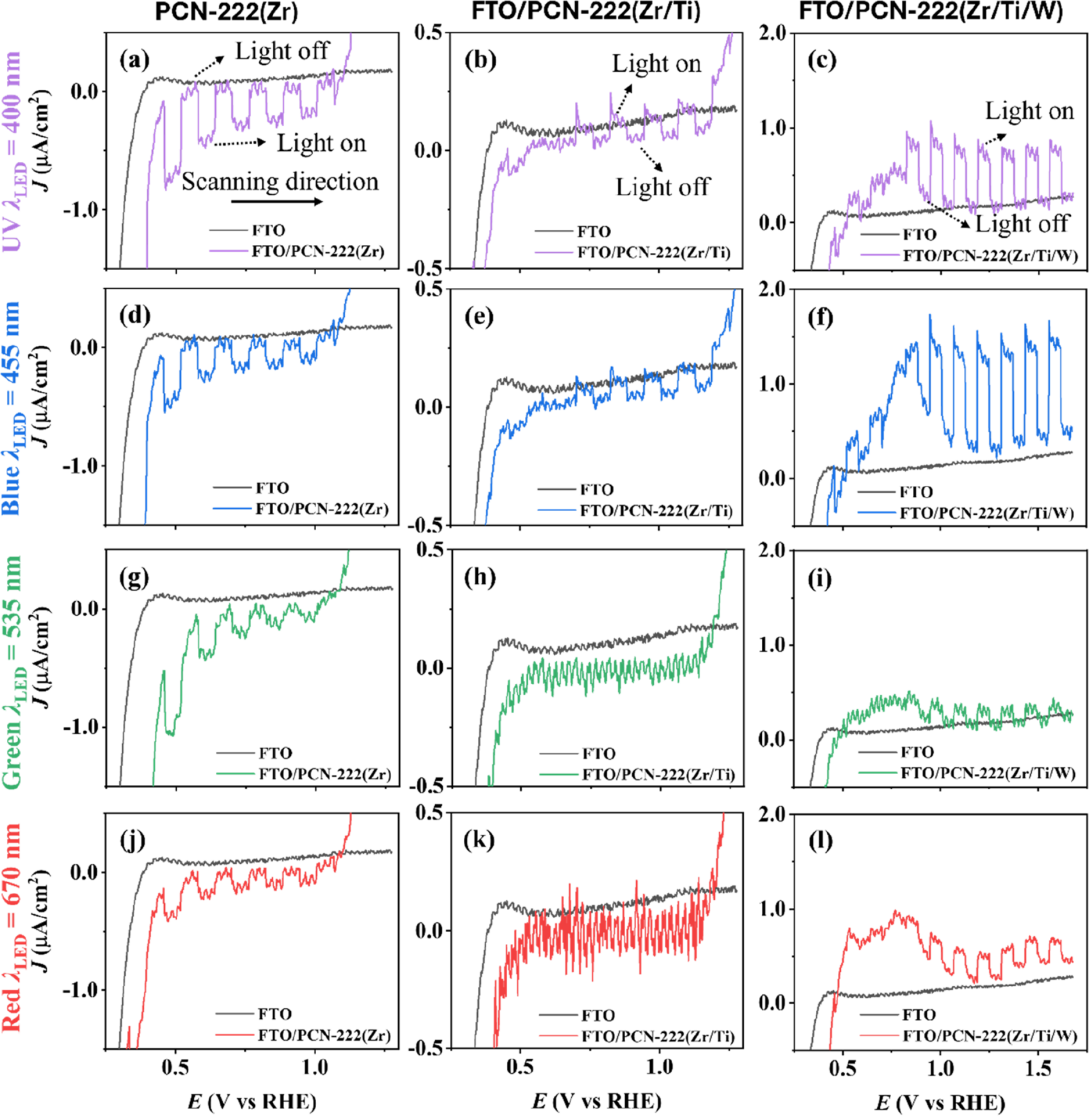
LSV curves with chopped front-side LED illumination under (a–c) λ_LED_ = 400 nm (UV), (d–f) λ_LED_ = 455 nm (blue), (g–i) λ_LED_ = 535 nm (green), and (j–l) λ_LED_ = 670 nm (red) at the same photon flux of 1.6 × 10^16^ cm^–2^ s^–1^ in 0.1 M acetate buffer as electrolyte with a scan rate of 20 mV s^–1^ for (a,d,g,j) FTO/PCN-222­(Zr), (b,e,h,k) FTO/PCN-222­(Zr/Ti), and (c,f,i,l) FTO/PCN-222­(Zr/Ti/W). The scan direction was from negative to positive potentials, and the potentials where the light was switched on and switched off, respectively, are indicated.

The FTO/PCN-222­(Zr) film acts as a photocathode, generating cathodic (negative) photocurrent with an onset potential of 0.45 V vs RHE. Under illumination, photogenerated electrons are transferred to the electrolyte, where two electrons participate in the reduction of water to hydrogen, as described by the water reduction half-reaction eq 1a. The photocurrent measured in the external circuit corresponds to the flux of photoelectrons to the solution, and photoholes to the FTO contact; the circuit is closed by the corresponding oxidation reaction at the counter electrode.

Upon incorporation of Ti and Ti/W into the PCN-222­(Zr) structure, the photoelectrochemical behavior changes significantly. The FTO/PCN-222­(Zr/Ti) and FTO/PCN-222­(Zr/Ti/W) photoelectrodes exhibit anodic (positive) photocurrent generation as photoanodes with onset potential at 0.70 and 0.80 V vs RHE, respectively. In this case, photogenerated holes are transferred to the electrolyte, driving the oxidation of water ideally to produce molecular oxygen, as described by the water oxidation half-reaction in eq 1b. In this case, the measured photocurrent corresponds to the net flux of photogenerated electrons to the FTO, and the flux of photoholes to the solution.^54^

1a $${2H}_{2}O_{\left(l\right)}+{2e}^{-}\to 2{OH}_{\left(aq\right)}^{-}+H_{2\left(g\right)}$$

1b $${2H}_{2}O_{\left(l\right)}+{4h}^{+}\to {4H}_{\left(aq\right)}^{+}+O_{2\left(g\right)}$$

All absorption bands of the measured porphyrin-based MOFs, including the Soret band (λ_max_ = 412 nm) and the four Q-bands (λ_max_ = 510–640 nm), are photoelectrochemically active, as evidenced by the photocurrent generation upon illumination at the respective wavelengths. The highest photocurrent response is obtained under LED illumination corresponding to the Soret band, highlighting its dominant contribution to the overall photoresponse. In contrast, under green and red LED illumination, the FTO/PCN-222­(Zr/Ti) electrode exhibits a noisy signal and negligible photocurrent, which may be attributed to limited charge separation efficiency at these wavelengths.

The FTO/PCN-222­(Zr/Ti/W) photoelectrodes (Figure 4c,f,i,l) appear to behave similarly to WO_3_ semiconductor electrodes, exhibiting a broad anodic feature centered at about 0.8 V vs RHE.^55,56^ The reoxidation potential, however, is markedly more positive than reported for WO_3_, where W^4+^ and W^5+^ centers were reoxidized at 0.18 and 0.38 V vs RHE, respectively. We attribute this positive shift, and the broader and less-defined peak, to the unique properties of the modified PCN-222­(Zr/Ti/W) composite material, resulting in an increase of the overpotential required to fully convert tungsten to W^6+^. This may be related to changes in local coordination and electronic structure associated with Ti/W incorporation within PCN-222­(Zr/Ti/W), which is supported by XPS.

Figure S12 presents the current density-potential (*J*–*E*) curves of FTO electrodes covered with thin oxide layers deposited by spray pyrolysis. The FTO/TiO_2_ electrodes were measured in a redox solution consisting of 0.01 M K_4_Fe­(CN)_6_ (Figure S12a) and 0.01 M K_3_Fe­(CN)_6_ (Figure S12b) in the 0.1 M acetate buffer. The FTO/NiO_*x*_ electrodes were evaluated in a nonaqueous solution of 0.01 M *p*-benzoquinone in propylene carbonate with 0.1 M TBAPF_6_ as inert electrolyte (Figure S12c). The absence of redox peaks for both FTO/TiO_2_ and FTO/NiO_*x*,_ thin films, in contrast to the results for bare FTO, indicates the compactness of both layers, preventing oxidation or reduction of the redox couple at uncovered FTO, thus confirming their role as compact blocking layers.

More importantly, beyond their blocking behavior, these thin oxide interlayers also serve as selective contacts that enhance the charge separation efficiency and lower recombination losses. TiO_2_ and NiO_*x*_ were selected due to their well-established use as charge-selective interfaces, particularly in hybrid perovskite solar cells,^57–62^ and their chemical stability and good compatibility with FTO substrates. TiO_2_ is an n-type semiconductor that may act as an electron transport layer (ETL), selectively extracting photogenerated electrons and blocking holes.^57–59^ Conversely, NiO_*x*_ is a p-type semiconductor that may function as a hole transport layer (HTL), enabling photogenerated hole extraction while suppressing electron transfer to FTO.^60–62^

Figure 5 shows the LSV curves for PCN-222­(Zr), PCN-222­(Zr/Ti), and PCN-222­(Zr/Ti/W) films spin-coated on FTO, FTO/TiO_2_, and FTO/NiO_*x*_, employed as electron- or hole-selective interlayers, respectively. Measurements were carried out in aqueous 0.1 M acetate buffer at a scan rate of 20 mV s^–1^ with chopped 1 sun AM1.5 G illumination. We observe a significant increase in photocurrent in all cases, which we attribute to enhanced charge separation efficiency and suppressed recombination due to the interfacial charge selectivity provided by the ETL (TiO_2_) and HTL (NiO_X_) layers.^57–62^ When TiO_2_ is used as the ETL, photocurrent enhancements of 5-, 10- and 6-fold are observed for PCN-222­(Zr), PCN-222­(Zr/Ti) and PCN-222­(Zr/Ti/W), respectively. In the case of NiO_*x*_ as the HTL, improvements of 7-, 4- and 3-fold are observed, respectively. Hence, the PCN-222 system demonstrates the interesting property of tunable photoelectrochemical polarity, acting either as a photoanode or a photocathode depending on the interfacial layer. This corresponds to either transferring electrons to FTO/TiO_2_ or holes to FTO/NiO_*x*_ related to the charge-extraction selectivity of the interlayer.

**Figure 5:**
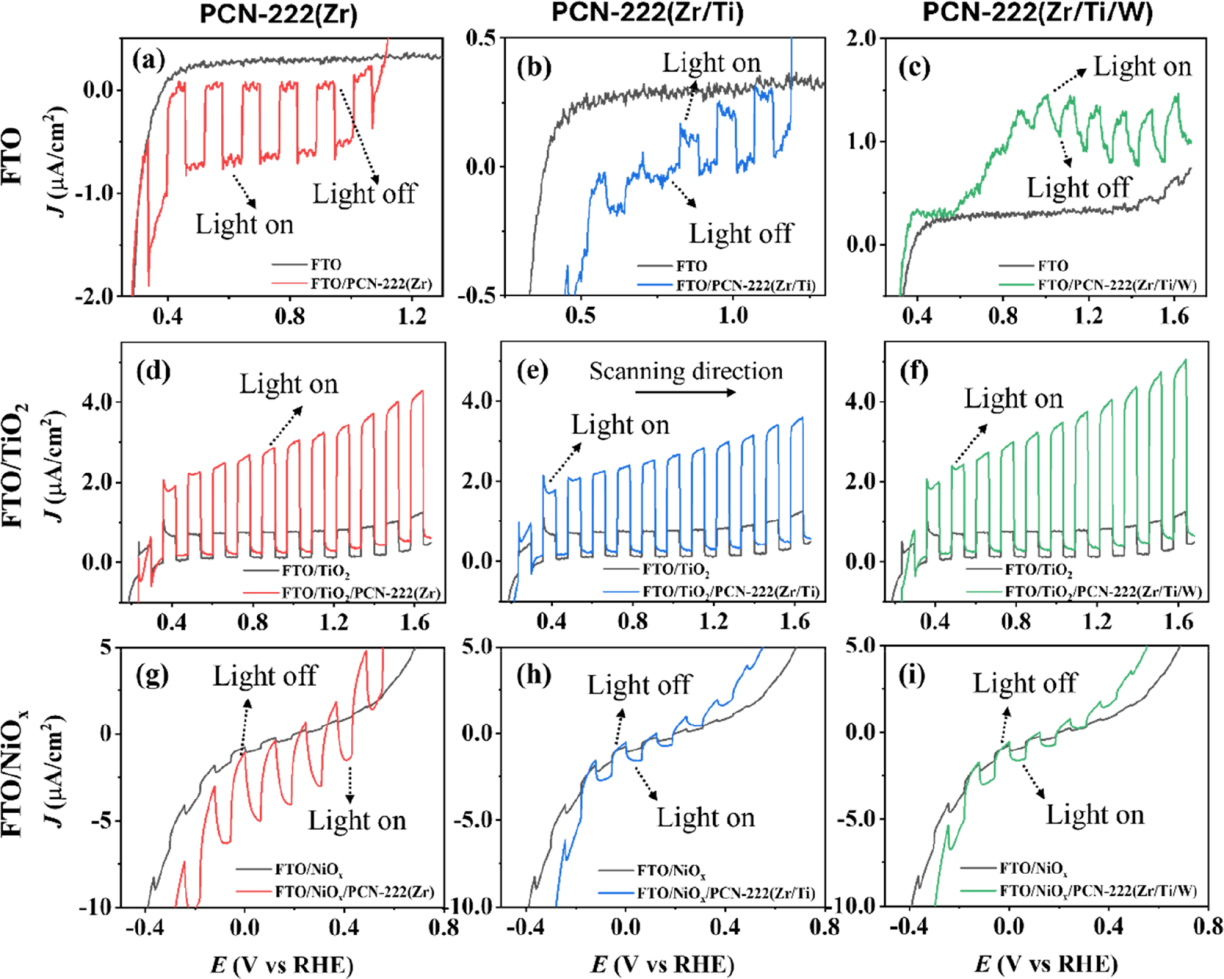
LSV curves with chopped front-side 1 sun AM 1.5G illumination in 0.1 M acetate buffer as electrolyte with a scan rate of 20 mV s^–1^ for (a,d,g) PCN-222­(Zr), (b,e,h) PCN-222­(Zr/Ti), and (c,f,i) PCN-222­(Zr/Ti/W) spin-coated on (a,b,c) FTO, (d,e,f) FTO/TiO_2_, and (g,h,i) FTO/NiO_*x*_ as different selective layers. The scan direction was from negative to positive potentials, and the potentials where the light was switched on and switched off, respectively, are indicated.

Notably, encapsulation of H_3_PW_12_O_40_ within the PCN-222­(Zr/Ti) framework further enhances the photocurrent by a factor of 2–5 (depending on the wavelength of illumination) when deposited on FTO. When deposited on FTO/TiO_2_ or FTO/NiO_*x*_, this enhancement is more modest (∼1.5-fold). The enhancement may be attributed to the presence of W centers, which may alter interfacial charge dynamics by introducing surface states or contributing to electrocatalytic activity. The electrocatalytic activity corresponds to improved charge transfer kinetics to the solution resulting in reduced recombination losses.^63–65^ Concomitantly, due to the effect of W-modification, the incorporation of the charge-selective interlayers results in a less-pronounced relative improvement.

Figure S13 schematically illustrates the characteristic shapes of the photocurrent transients shown in Figure 5, which are applicable to both photoanode and photocathode behaviors, but with opposite photocurrent polarities. The initial photocurrent spike observed upon illumination corresponds to the fast separation of photogenerated electron–hole pairs. For a photocathode, electrons are driven toward the electrolyte interface, while holes are collected in the external circuit. In the case of FTO/PCN-222­(Zr), the photocurrent under illumination reaches a steady-state condition without significant losses, resulting in a rectangular-shaped transient. This shape is characteristic for the situation where recombination does not occur on the time scale of the measurement (see Figure S13a). A similar transient behavior is observed for FTO/TiO_2_/PCN-222 at applied potentials more positive than 0.5 V vs RHE. However, in this case, as the steady-state hole photocurrent increases with potential, the rectangular shape becomes deformed (see Figure S13b).

For FTO/NiO_*x*_/PCN-222 and FTO/PCN-222­(Zr/Ti/W), a slow increase in photocurrent is observed upon illumination until steady-state is reached, followed by a slow decay after switching off the light. This behavior is indicative of electron trapping during illumination. When the light is switched on, photogenerated electrons first fill trap states within the material before a measurable current appears. The density of such traps, which often depends on the applied potential and light intensity, can be estimated from the “missing” current and the time required to reach steady-state conditions. Upon light off, the gradual release of trapped charges occurs as the Fermi level slowly returns to equilibrium (see Figure S13c).

For PCN-222­(Zr/Ti), and especially for FTO/TiO_2_/PCN-222 at low applied potential, a photocurrent peak and subsequent decay is observed before reaching steady-state conditions, followed by an overshoot of the same intensity but opposite sign upon turning the light off. This transient behavior can be attributed to the accumulation of photogenerated holes at or near the electrolyte interface, which promotes interfacial recombination, resulting in a decrease in the net photocurrent. However, if the overshoot upon light-off is absent or differs in magnitude compared to the light-on, it may indicate that accumulation of holes at the interface alters the potential distribution at the electrolyte junction, as observed at more positive potentials (see Figure S13d).^25,66–73^ Note that these current vs potential measurements are not suitable for the determination of recombination kinetics, which can be obtained from current vs time transients or impedance or intensity-modulated photocurrent spectroscopy.

### 3.4 Band Alignment and Mechanistic Interpretation of Photocurrent Directionality

Figure 6 presents a schematic illustration of the charge carrier dynamics under illumination for the different electrode configurations, along with the corresponding band alignments relative to the water redox potentials. The diagrams illustrate the processes of photogeneration, charge separation, and transport of photogenerated electrons and holes at the electrode–electrolyte interface. In all configurations, PCN-222 serves as the principal photoactive layer, while TiO_2_ and NiO_x,_ although capable of absorbing light in the UV region and generating photocurrent (as shown in Figure 5d–i), mainly function as electron and hole extraction and transport layers (ETL and HTL), respectively.

**Figure 6:**
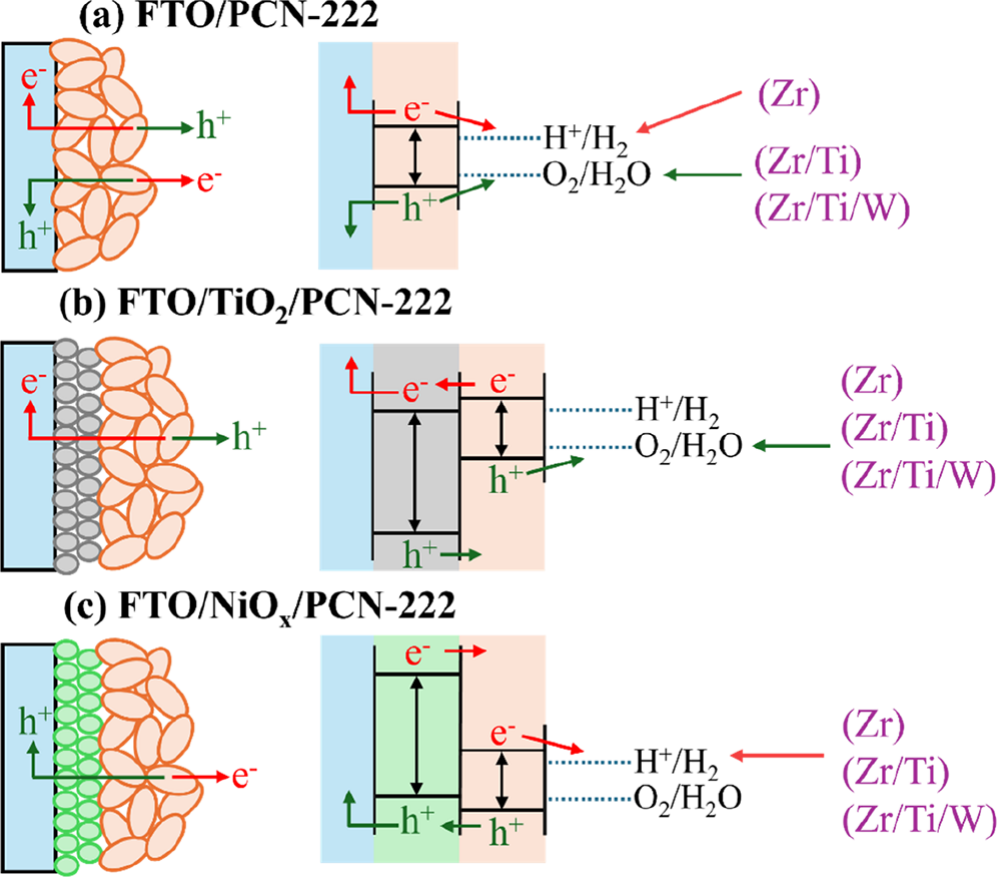
Schematic representation of charge carriers dynamics under illumination and in contact with an electrolyte for (a) FTO/PCN-222, (b) FTO/TiO_2_/PCN-222, and (c) FTO/NiO_*x*_/PCN-222. FTO acts as a nonselective contact, while TiO_2_ and NiO_*x*_ function as electron- and hole-transport layers (ETL and HTL), respectively.

In the FTO/PCN-222 configuration (Figure 6a), photoinduced electron–hole pairs are generated within the PCN-222 material, with FTO acting as a nonselective contact. Depending on the specific PCN-material deposited on the FTO, the electrode behaves either as a photocathode (when using pristine PCN-222­(Zr)) or as a photoanode (when using PCN-222­(Zr/Ti) and PCN-222­(Zr/Ti/W), promoting water reduction or oxidation, respectively.

Applying the postsynthetic metal node exchange method reported here to produce Ti- and W-modified PCN-222­(Zr) materials significantly alters the electronic and optical properties of the pristine framework. The incorporation of Ti leading to mixed Zr/Ti-oxo nodes, the formation of Ti-rich surface domains as discussed above, and the encapsulation of H_3_PW_12_O_40_ within the mesopores all contribute to an increased local electron density within the framework. These modifications influence the band structure through changes in the coordination environment of the metal ions, as supported by XPS, and may introduce new surface states, thereby affecting the optical transitions and charge carrier dynamics. Optical characterization reveals a bandgap narrowing upon Ti- and W-modification, likely due to shifts in the conduction or valence band edge. Given that the band edge positions in PCN-222­(Zr) are close to the redox potentials of water (Figure 6a), even slight shifts induced by Ti-/W-modification can alter the alignment with respect to the water oxidation or reduction levels. This alignment is critical in determining the thermodynamic favorability of the redox processes and, consequently, the direction of photoinduced charge transfer.^18,65,74^ Indeed, the negative shift of Zr 3d binding energy upon Ti incorporation, together with the positive shifts of both Zr and Ti signals after POM encapsulation (indicated by XPS), highlight the electronic perturbations at the metal–oxo clusters, which correlate with the polarity switching and photocurrent enhancement observed in the photoelectrochemical experiments. It should be noted that the band alignment is expected to depend on the surface chemistry of both the ETL/HTL and the PCN-222 materials, which in turn depends on the applied potential and illumination conditions; hence, at this point we have not been able to determine band edge positions under working conditions, but rather focus on the charge extraction characteristics.

An additional explanation for the change in photocurrent polarity may involve the electrocatalytic role of the incorporated Ti and W species. Ti and W are known as photoanodic materials for water oxidation, and their presence at the PCN-222/electrolyte interface may generate catalytically active sites or induce surface states that lower the kinetic barrier for hole transfer. This change in the semiconductor/electrolyte interface selectivity ultimately determines the direction of the photocurrent flow. In the FTO/TiO_2_/PCN-222 system (Figure 6b), the TiO_2_ layer improves charge separation by efficiently extracting photogenerated electrons from PCN-222 and transporting them toward the FTO back contact. This reduces charge recombination and promotes hole transfer to the electrolyte, thereby favoring oxidation reactions. Notably, this strategy is effective even for PCN-222­(Zr) which, when deposited directly on FTO, operates as a photocathode. Conversely, in the FTO/NiO_*x*_/PCN-222 configuration (Figure 6c), the NiO_*x*_ layer selectively extracts photogenerated holes from the PCN-222 photoactive layer, transporting them toward the FTO substrate. This facilitates electron transfer from PCN-222 to the electrolyte, thus promoting reduction reactions. As a result, this architecture reinforces the photocathodic response not only for PCN-222­(Zr) but also for PCN-222­(Zr/Ti) and PCN-222­(Zr/Ti/W), regardless of their intrinsic photoelectrochemical behavior when deposited on bare FTO.

Hence, these results indicate that the overall photoelectrochemical behavior is governed not by the doping density and dopant properties of the PCN-222 material, which behaves as an intrinsic semiconductor, but rather by the selectivity of the contact with the external circuit. While differences in oxidation and reduction kinetics at the electrolyte interface could, in principle, affect the direction of current flow, our findings clearly show that the nature of the contact at the FTO substrate plays a dominant role in determining the photoelectrochemical properties and the photoresponse polarity.

In addition, the incorporation of Ti- and W- within the PCN-222­(Zr) structure may further modulate this behavior through their potential electrocatalytic activity. In particular, the phosphotungstate material incorporated in the mesopores exhibits redox reactions involving W^4+^ and/or W^5+^ centers, and upon applying a more positive potential, the reoxidized POM improves hole extraction, thus imposing selectivity at the electrolyte interface and reducing the impact of the oxide interlayer. The ability to switch the photoelectrode response from photocathodic to photoanodic (or vice versa) enables the same MOF-based materials to be selectively operated under either reductive or oxidative conditions. This polarity control is particularly relevant for photoelectrochemical systems where the target reaction depends on the dominant charge carrier, allowing the electrode function to be adapted through interfacial engineering rather than material replacement.

## 4 Conclusions

This work demonstrates a stepwise strategy to modulate the photoelectrochemical properties of the mesoporous PCN-222 through a combination of metal-node substitution and pore encapsulation, followed by integration into functional photoelectrodes with charge-selective extraction layers. Ti was successfully incorporated into the framework via postsynthetic metal node exchange, preserving the crystallinity and nanorod morphology, while introducing lattice distortion and band structure modification. Subsequent encapsulation of the polyoxometalate H_3_PW_12_O_40_ within the PCN mesopores further altered the optical properties and enhanced photocurrent generation. These chemical modifications collectively shifted the photoelectrode behavior from photocathodic to photoanodic under visible light illumination, attributed to changes in band edge positions and electrocatalytic contributions. Moreover, the directionality of the photocurrent was found to be strongly dependent on the nature of the interfacial layer (TiO_2_ or NiO_*x*_), overriding the intrinsic semiconductor character of the PCN-222 and enabling tunable photoelectrode architectures. This polarity control highlights the potential of PCN-222-based electrodes for versatile photoelectrochemical configurations, where charge directionality can be tailored to match specific reaction environments. These findings provide valuable insights into the design of multicomponent MOF-based systems with tailored optoelectronic properties and open new avenues for their application in photoelectrochemical devices.

## Supplementary Material



## Data Availability

Data for this article, including the data used for all figures in the manuscript and the Supporting Information, are available at Zenodo at 10.5281/zenodo.18088960

## Notes

Contains detailed EDX mapping and DLC results, photographs of the materials dispersions and photoelectrodes, XPS spectra, absorbance and reflectance spectra, Tauc and Kubelka–Munk graphs and analysis, current–potential curves of K_4_Fe­(CN)_6_ and benzoquinone, and analysis of the time-dependent current–potential curves under illumination (PDF)
