# Supplementary material for: Tuning the Photoelectrochemical Properties of Ti/W-Modified PCN-222 Using Charge–Selective Interfaces
Source: ACS Appl Mater Interfaces. 2026 Jan 16;18(6):9877–91. doi: 10.1021/acsami.5c22732 (PMC12926951; doi:10.1021/acsami.5c22732)
Supplement: Supplementary file 1 [file am5c22732_si_001.pdf]

SUPPORTING INFORMATION

Tuning the Photoelectrochemical Properties of Ti/W-Modified PCN-222 Using Charge-Selective Interfaces

Juan Carlos Expósito-Gálvez[1], Florencia Vattier[2], José María Pedrosa[1], Carolina Carrillo-Carrión[3*], Gerko Oskam[1*]

[1]*Center for Nanoscience and Sustainable Technologies (CNATS). Department of Physical, Chemical and Natural Systems, Universidad Pablo de Olavide, 41013, Seville, Spain*

[2]*Department of Inorganic Chemistry, and Center for Innovation in Advanced Chemistry (ORFEO-CINQA). Institute for Chemical Research (IIQ), CSIC-University of Seville, 41092, Seville, Spain*

[3]*Institute for Chemical Research (IIQ), CSIC-University of Seville, 41092, Seville, Spain*

Corresponding Authors: gosk@upo.es; carolina.carrillo@csic.es

Table S1. EDX analyses of the samples, showing the atomic percentage (at.%) of Zr, Ti, and W, the corresponding atomic ratios, and the calculated amounts per Zr_6 cluster.

Sample	Zr (at.%)	Ti (at.%)	W (at.%)	Ti/Zr ratio	Ti per Zr_6	PW_{12} per Zr_6
PCN-222(Zr)	100	-	-	-	-	-
PCN-222(Zr/Ti)	51.9	48.1	-	0.927	5.54	-
PCN-222(Zr/Ti/W)	32.4	28.6	38.9	0.883	5.30	0.6

Table S2. Hydrodynamic length L_h (mean value ± SD) derived from intensity distributions and *ζ-potential* (mean value ± SD) of the different PCN-222 materials dispersed in methanol (for DLS) and water (for *ζ-potential*). The polydispersity index (*PDI*) is also given.

Sample	L_h (nm)	*PDI*	*ζ-potential* (mV)
PCN-222(Zr)	138.5 ± 0.2	0.061	27.2 ± 0.3
PCN-222(Zr/Ti)	149.5 ± 1.4	0.102	34.6 ± 0.3
PCN-222(Zr/Ti/W)	160.9 ± 0.9	0.098	31.8 ± 0.9

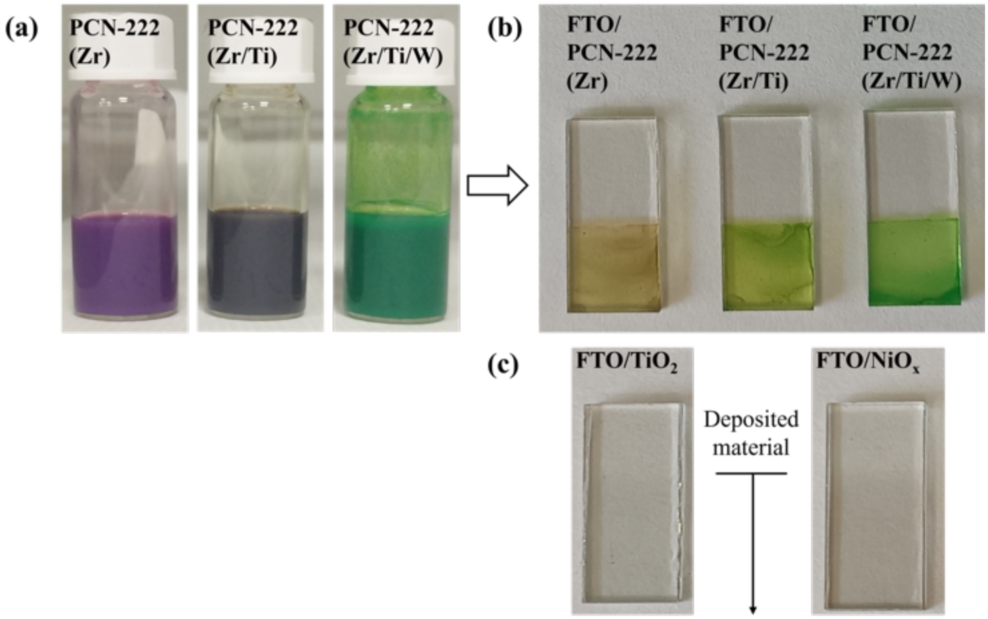

Figure S1. (a) Photographs of PCN-222(Zr), PCN-222(Zr/Ti), and PCN-222(Zr/Ti/W) dispersed in methanol (5 mg mL^{-1}). (b) Photographs of the photoelectrodes prepared by deposition of the different PCN-222 material on FTO by spin coating technique, and (c) compact layers of TiO_2 and NiO_x deposited on FTO by spray pyrolysis.

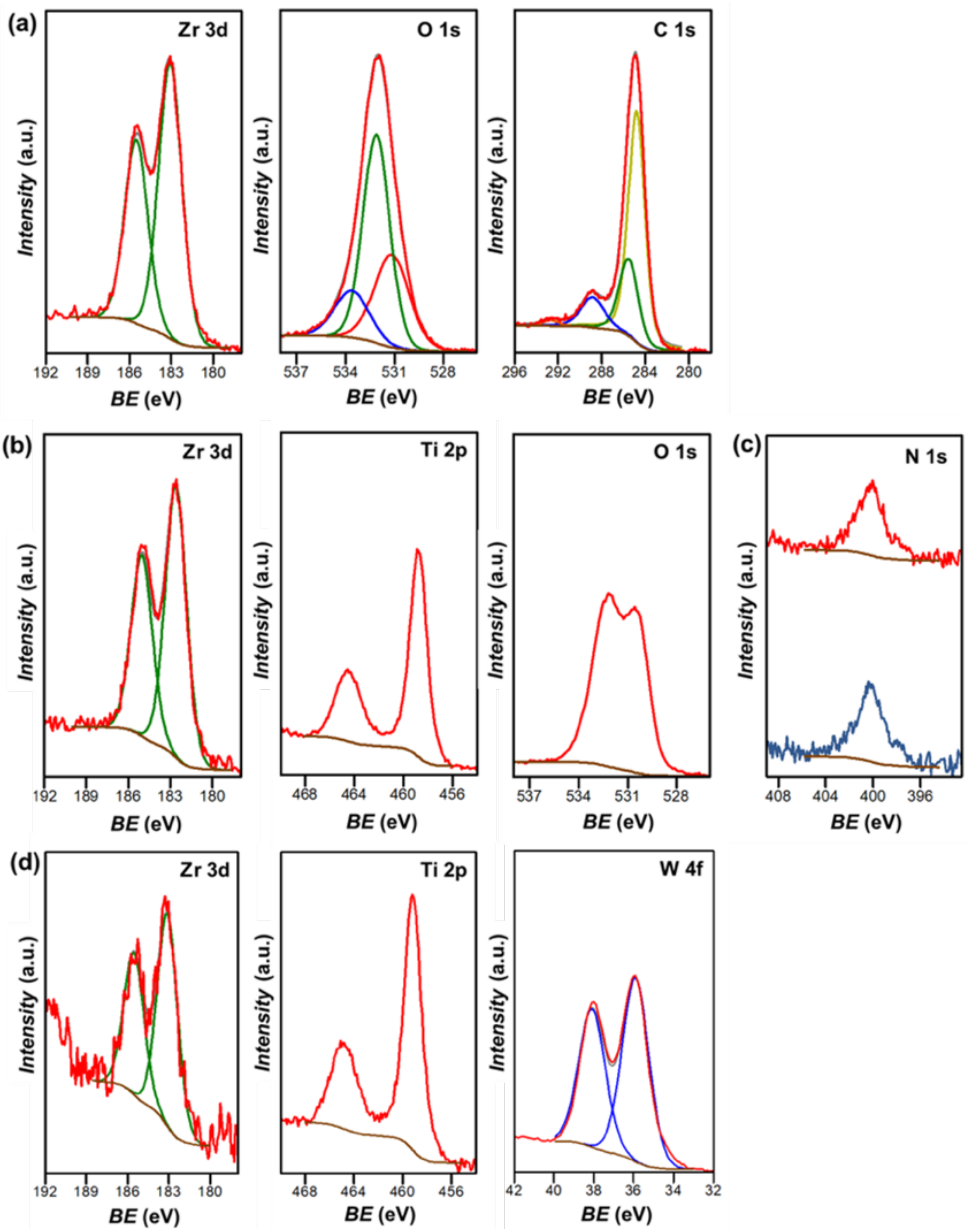

Figure S2. (a) Zr 3d, O 1s and C 1s XPS spectra for PCN-222(Zr) sample. (b) (c) Zr 3d, Ti 2p and O 1s XPS spectra for PCN-222(Zr/Ti) sample. (c) Comparison N 1s region XPS spectra of PCN-222(Zr) (top), and PCN-222(Zr/Ti) (bottom). (d) Zr 3d, Ti 2p and W 4f XPS spectra for PCN-222(Zr/Ti/W) sample.

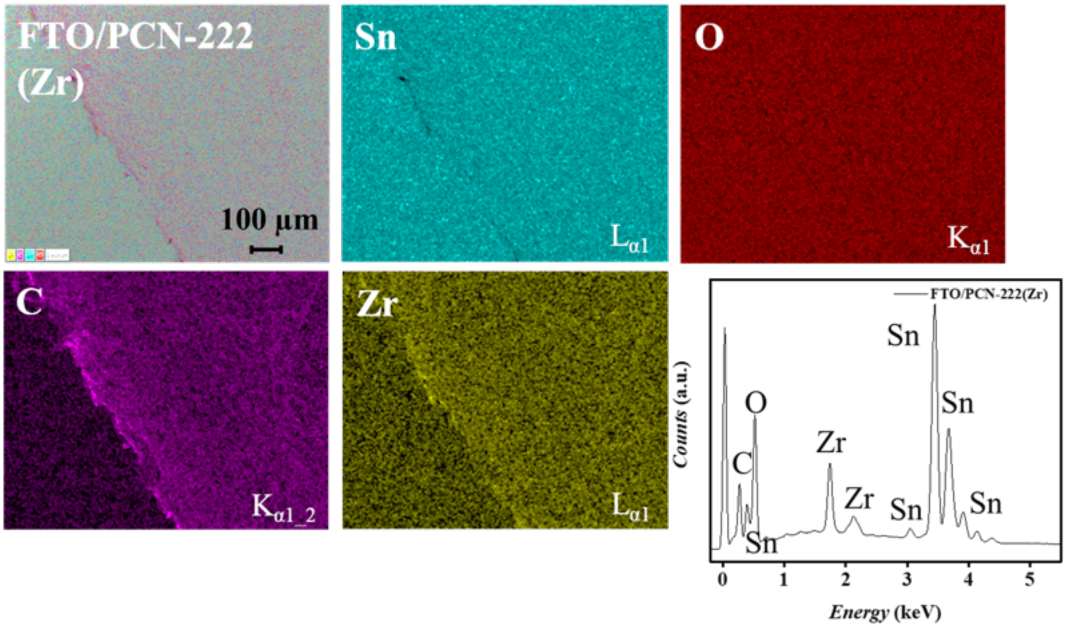

Figure S3. EDX mapping of PCN-222(Zr) layer deposited by spin coating on FTO.

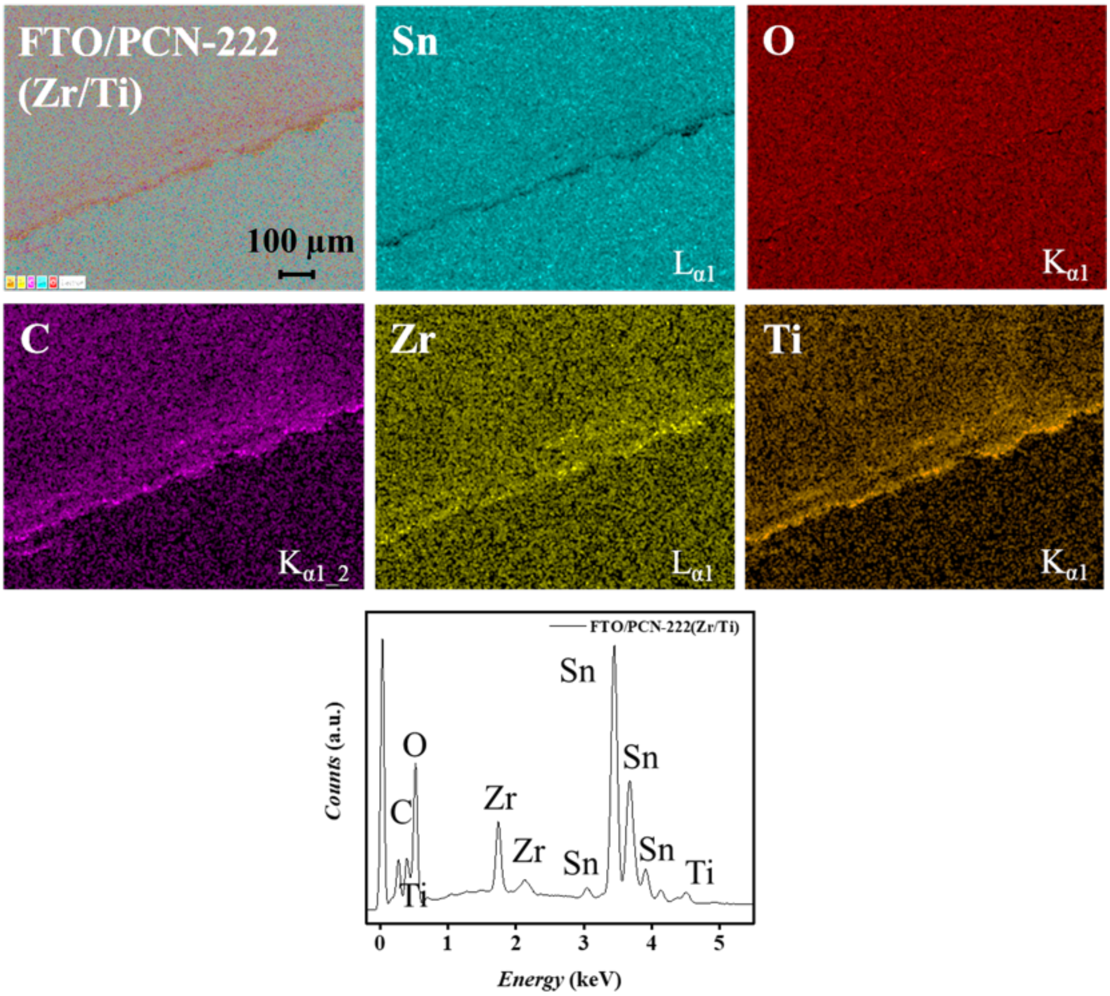

Figure S4. EDX mapping of PCN-222(Zr/Ti) layer deposited by spin coating on FTO.

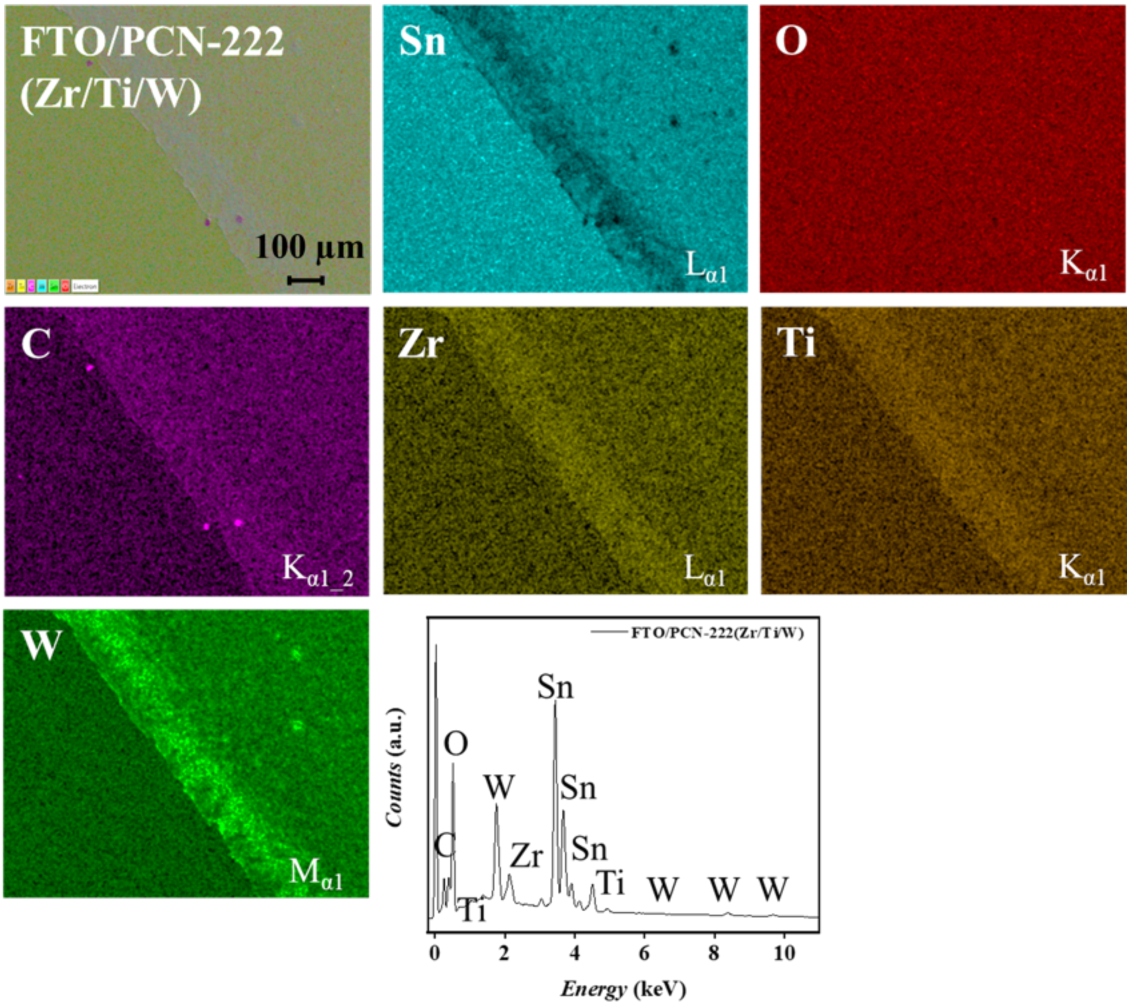

Figure S5. EDX mapping of PCN-222(Zr/Ti/W) layer deposited by spin coating on FTO.

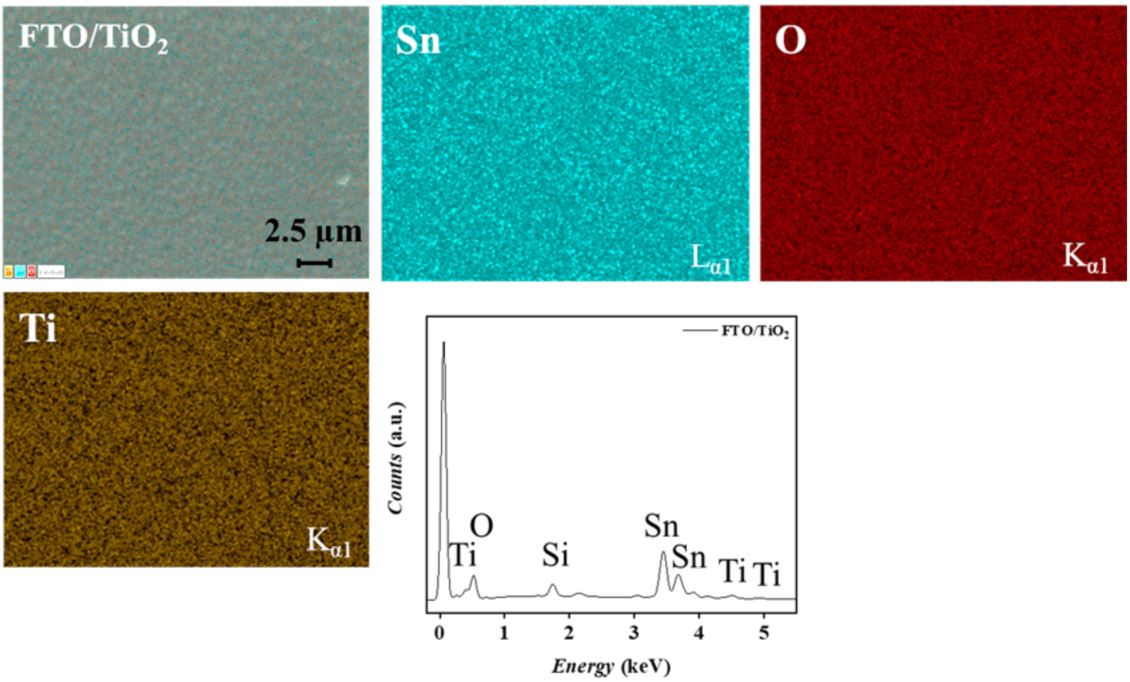

Figure S6. EDX mapping of the TiO_2 layer deposited by spray pyrolysis on FTO.

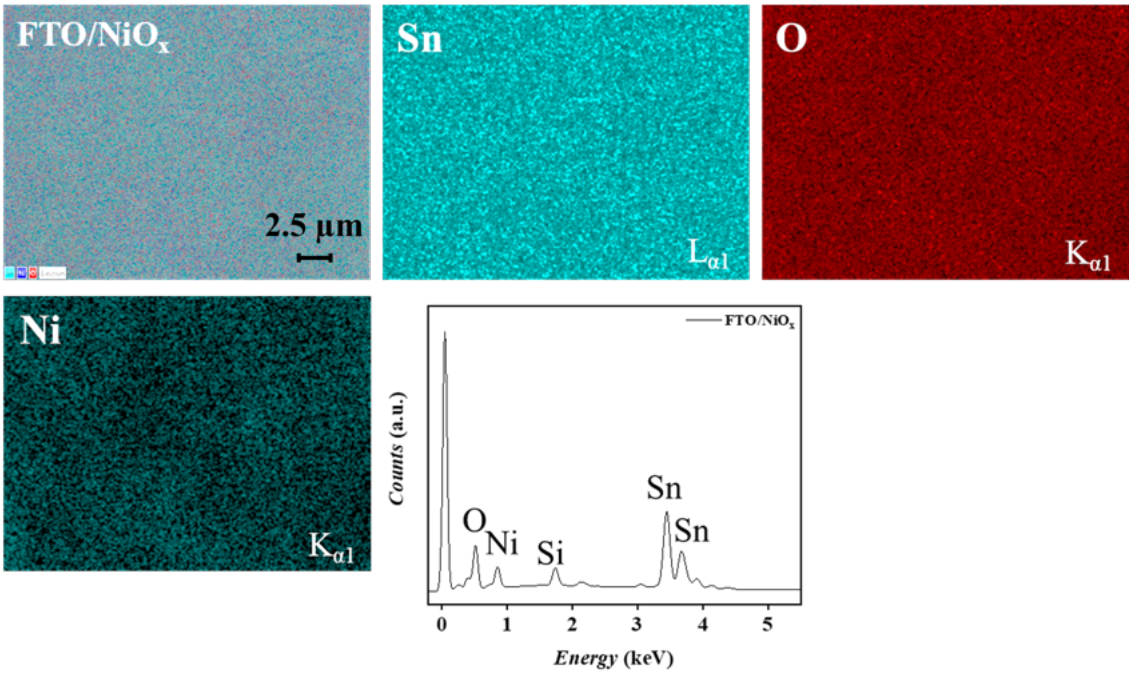

Figure S7. EDX mapping of the NiO_x layer deposited by spray pyrolysis on FTO.

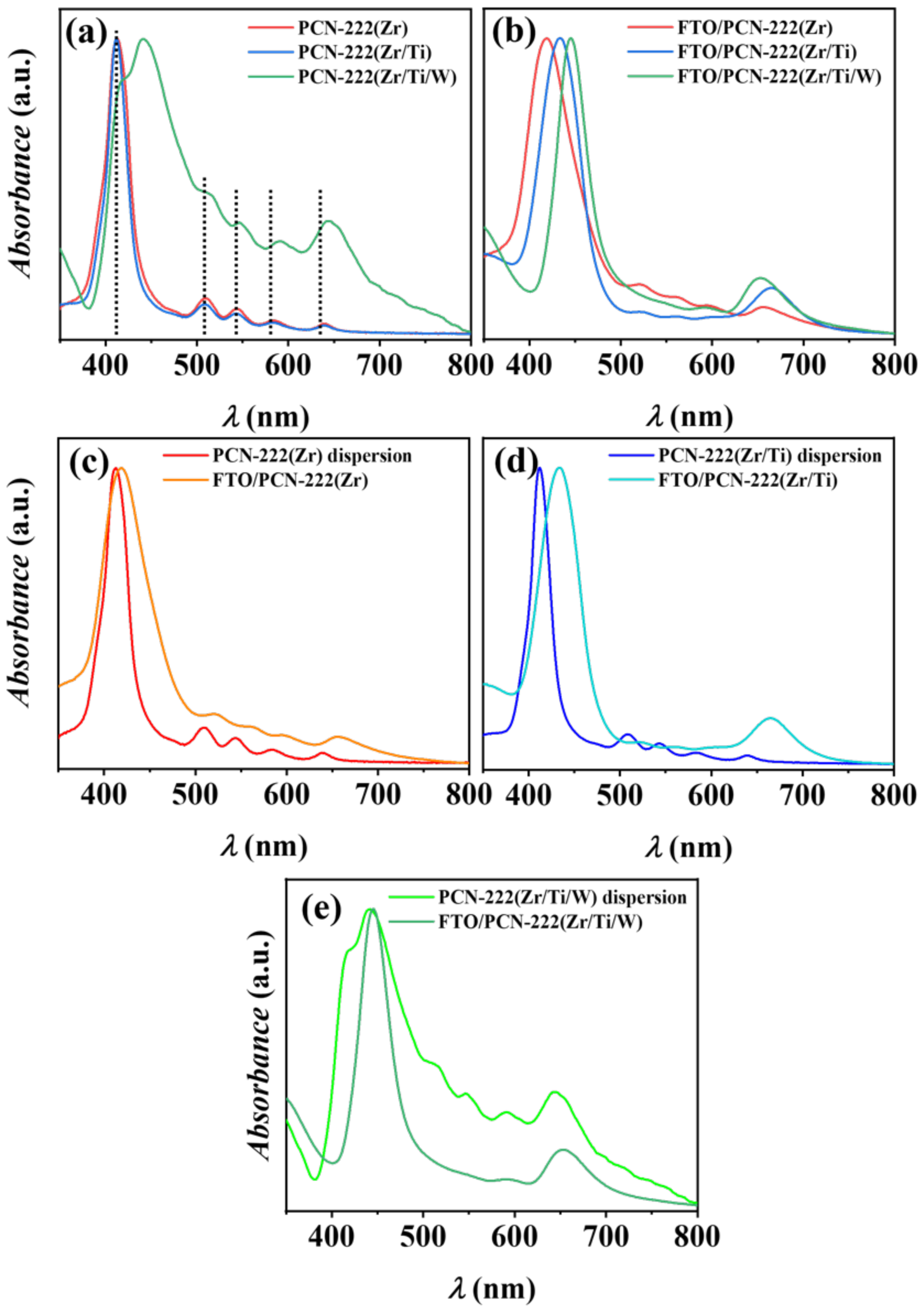

Figure S8. Absorbance spectra of (a) dispersed PCN-222 with a concentration of 0.03 mg/mL in methanol, and (b) FTO/PCN-222 electrode. Comparison between dispersed (light color) and deposited on FTO (dark color) of (c) PCN-222(Zr), (d) PCN-222(Zr/Ti), and (e) PCN-222(Zr/Ti/W).

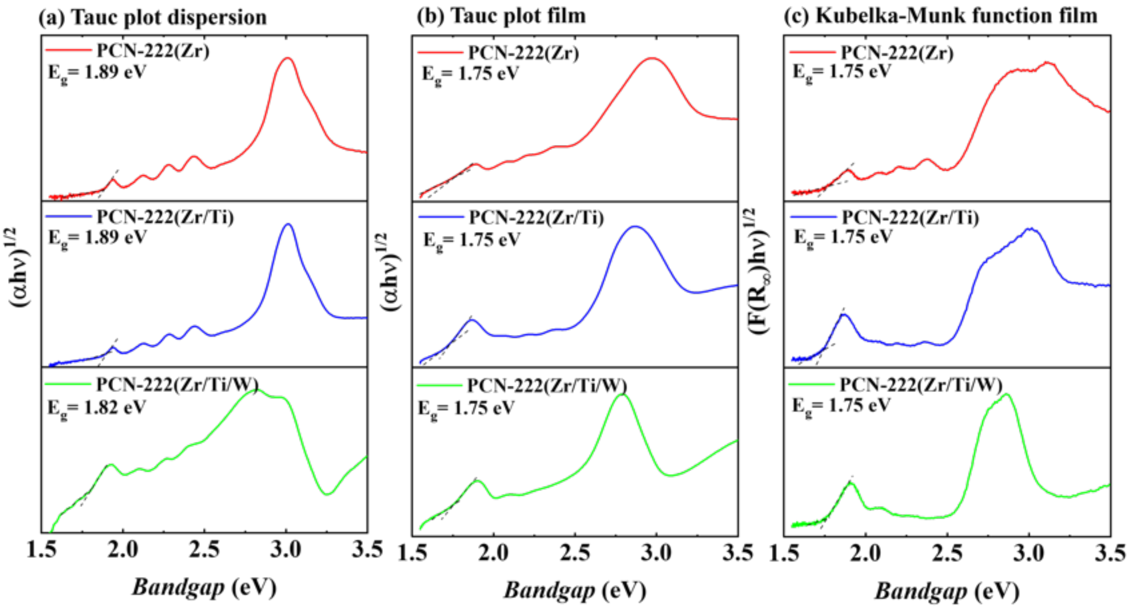

Figure S9. Indirect bandgap estimation of (a) dispersed PCN-222 with a concentration of 0.03 mg mL^{-1} in methanol and (b,c) spin-coated on FTO, using different estimation methods as (a,b) Tauc plot from absorbance spectroscopy and (c) Kubelka-Munk analysis from diffuse reflectance spectroscopy.

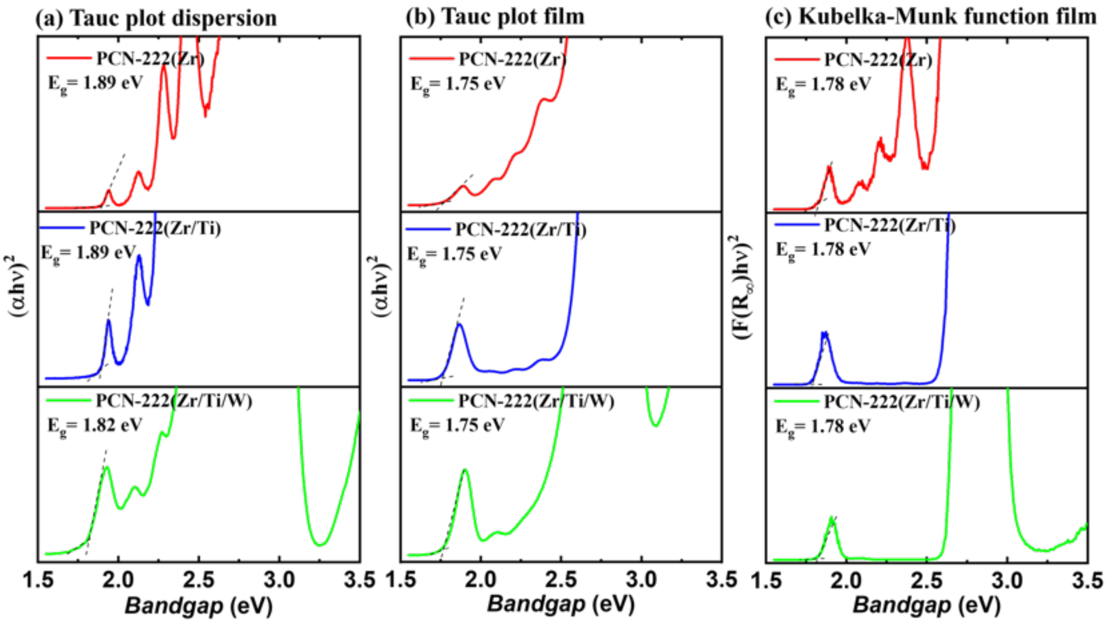

Figure S10. Direct bandgap estimation of (a) dispersed PCN-222 with a concentration of 0.03 mg mL^{-1} in methanol and (b,c) spin-coated on FTO, using different estimation methods as (a,b) Tauc plot from absorbance spectroscopy and (c) Kubelka-Munk analysis from diffuse reflectance spectroscopy.

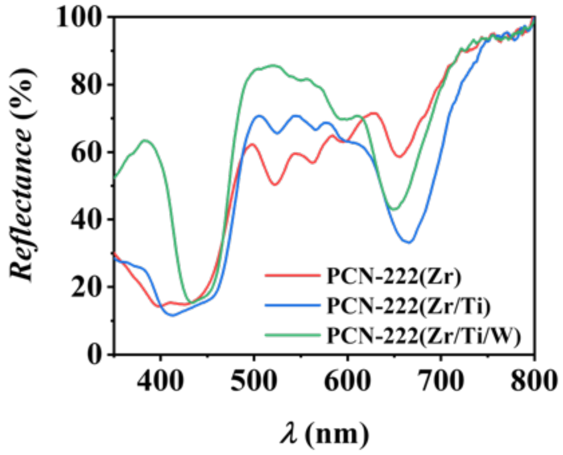

Figure S11. Diffuse reflectance spectroscopy of FTO/PCN-222(Zr), FTO/PCN-222(Zr/Ti), and FTO/PCN-222(Zr/Ti/W), electrodes measured using an integration sphere.

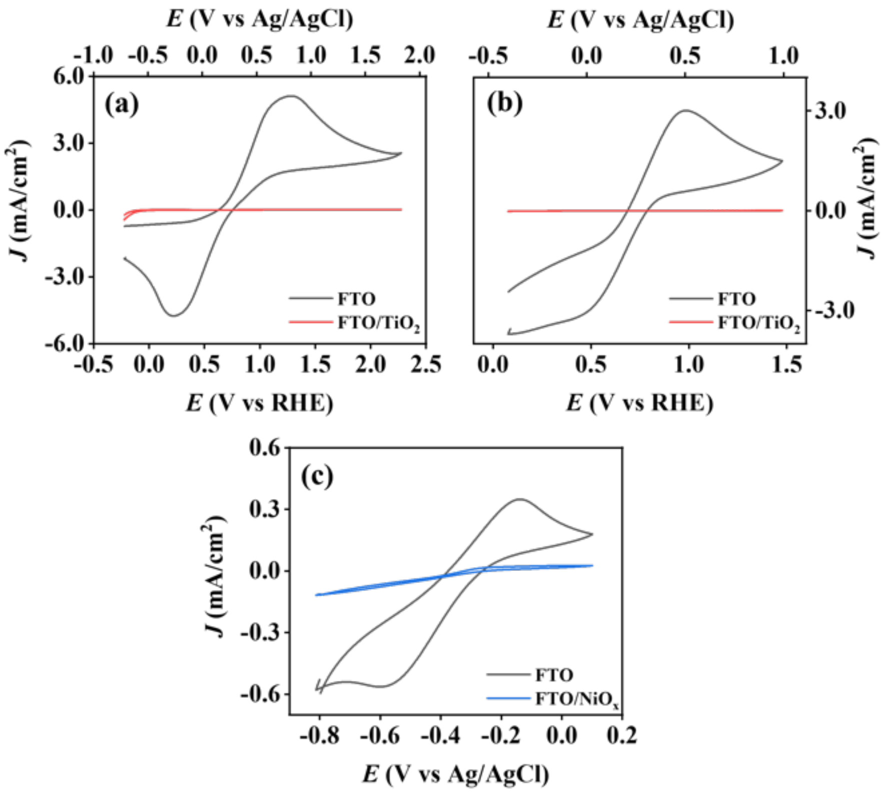

Figure S12. *J-E* curves under dark conditions at scan rate of 20 mV s^{-1} of thin films (a) FTO/TiO_2 with 0.1 M acetate buffer with 0.01 M $[Fe(CN)_6]^{4-}$ and (b) FTO/TiO_2 with 0.1 M acetate buffer with 0.01 M $[Fe(CN)_6]^{3-}$ both at pH=4.5 as electrolytes, and (c)FTO/NiO_x with non-aqueous solution of 0.1 M propylene carbonate with 0.1 M $TBAPF_6$ with 0.01 M p-benzoquinone as electrolyte. Both films deposited by spray pyrolysis on FTO and compared with bare-FTO.

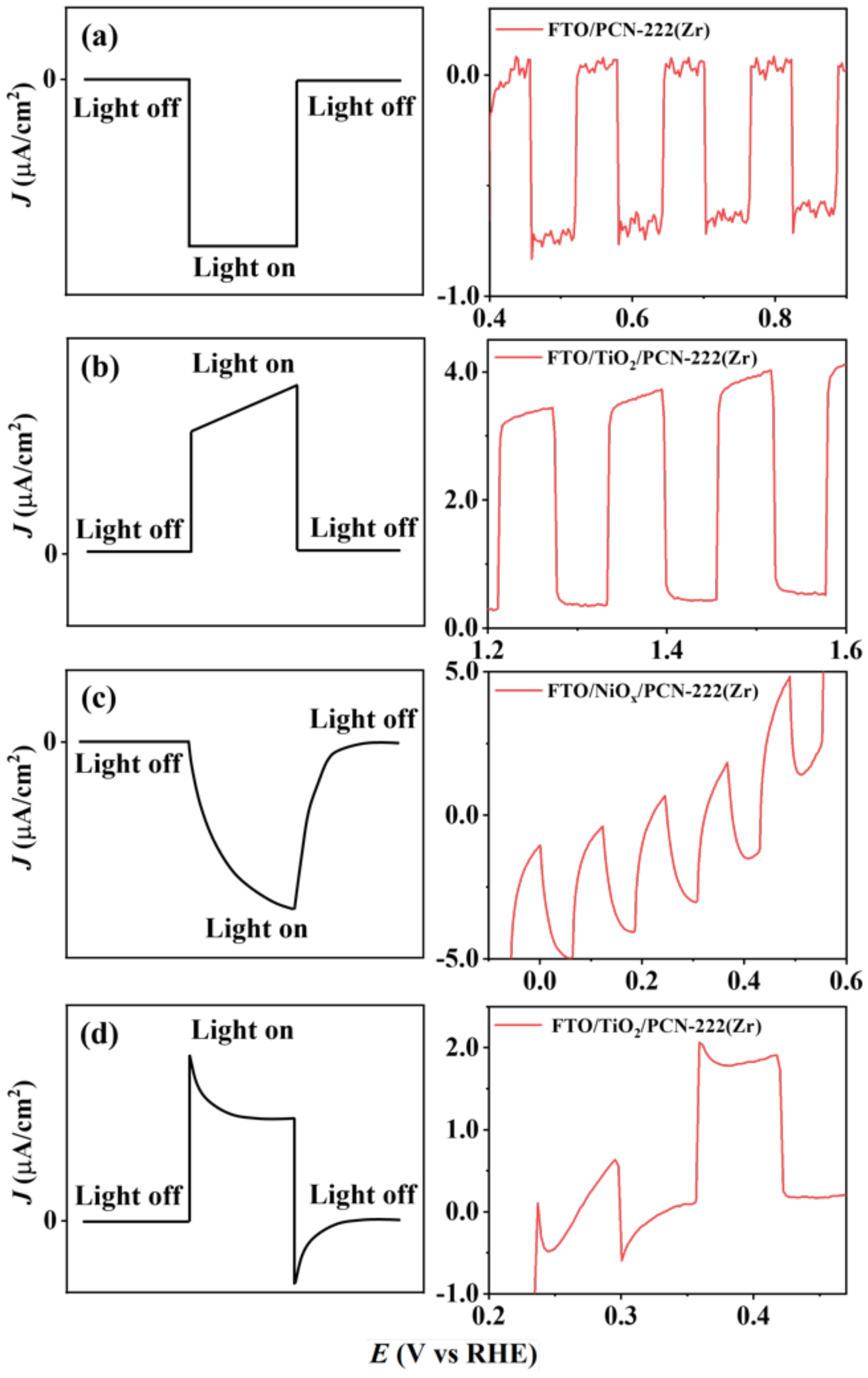

Figure S13. Schematic of the origin of transient photocurrent responses under chopped illumination, along with the representative experimental behaviors. (a) Electron (for photocathode) photocurrent without surface recombination, (b) hole (for photoanode) photocurrent increasing the applied potential, (c) surface electron-hole recombination due to accumulation of hole (for photoanode) at the surface, inducing band edge unpinning, (d) surface trapped electron (for photocathode). This schematic is valid for both photoanode and photocathode behaviors with opposite photocurrent signals (positive photocurrent for photoanode and negative photocurrent for photocathode).
